# Structural Mechanisms of Quasi-2D Perovskites for Next-Generation Photovoltaics

**DOI:** 10.1007/s40820-024-01609-9

**Published:** 2025-02-08

**Authors:** Hyeonseok Lee, Taeho Moon, Younghyun Lee, Jinhyun Kim

**Affiliations:** 1 Department of Chemistry, Kwangwoon University, Seoul, 01897 Republic of Korea; 2 Department of Materials Science and Engineering, Dankook University, Cheonan, 31116 Republic of Korea; 3 Center for Semiconductor Technology, Korea Institute of Science and Technology (KIST), Seoul, 02792 Republic of Korea

**Keywords:** Perovskite, Dion-Jacobson, Ruddlesden-Popper, Quantum structure, Quasi-2D perovskite

## Abstract

Quasi-two-dimensional (2D) perovskite embodies characteristics of both three-dimensional (3D) and 2D perovskites, achieving the superior external environment stability structure of 2D perovskites alongside the high efficiency of 3D perovskites. This effect is realized through critical structural modifications in device fabrication. Typically, perovskites have an octahedral structure, generally ABX_3_, where an organic ammonium cation (A') participates in forming the perovskite structure, with A'_(n)_ (n = 1 or 2) sandwiched between A_(n-1)_B_(n)_X_(3n+1)_ perovskite layers. Depending on whether A' is a monovalent or divalent cation, 2D perovskites are classified into Ruddlesden-Popper perovskite or Dion-Jacobson perovskite, each generating different structures. Although each structure achieves similar effects, they incorporate distinct mechanisms in their formation. And according to these different structures, various properties appear, and additive and optimizing methods to increase the efficiency of 3D perovskites also exist in 2D perovskites. In this review, scientific understanding and engineering perspectives of the quasi-2D perovskite is investigated, and the optimal structure quasi-2D and the device optimization is also discussed to provide the insight in the field.

## Highlights

- This review highlights the structural advantages and challenges of qausi-2D perovskite.
- Beyond these structural adaptations, unique additive methods specific to quasi-2D perovskites are suggested, alongside future directions for further improvement.
- Material and device analysis using Ruddlesden–Popper, Dion–Jacobson, and alternating cation phases are discussed.

## Graphical abstract

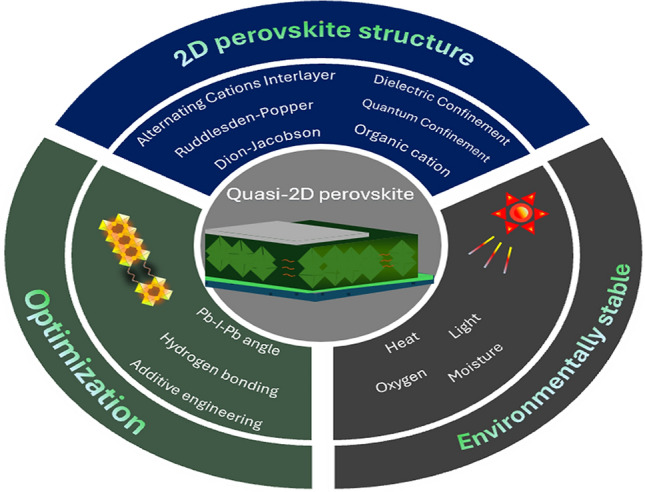

## Introduction

Perovskites have gained significant attention in the semiconductor field due to their ability to form thin films through solution process from precursors, making the fabrication process both cost-effective and straightforward [1–4]. Over the past few decades, perovskite technology has advanced rapidly, achieving a power conversion efficiency (PCE) of 26.7% in solar cells [5]. With remarkable advancements in the field of perovskite solar cells (PSCs), perovskites have potential in various applications such as light-emitting diodes (LEDs), thin-film field-effect transistors (TFTs), and image sensors [6–18]. Additionally, the tunability of the bandgap allows perovskites to absorb a wide range of wavelengths, facilitating the development of high-efficiency tandem solar cells by integrating perovskites with both narrow and wide bandgaps [19–26].

However, these advantages are significantly undermined by the vulnerability of perovskites to ambient environmental conditions. The most widely used perovskites are organic halide perovskites (OHPs) [27, 28], typically composed of an organic cation (A), a metal cation (B), and a halide anion (X). The metal cations (B) are hydrophilic and highly susceptible to moisture in the air, while the halide anions are prone to ion migration under light exposure. These stability issues related to moisture and light significantly reduce the efficiency and lifetime of perovskite devices, posing substantial challenges to their commercialization [29–33].

To address these stability challenges, extensive research has focused on inserting organic ammonium cations (A') as spacers within the ABX_3_ structure. These A' cation spacers act as barriers, reducing exposure to the external environment and increasing exciton binding energy through the quantum well (QW) effect [34–39]. This enhances both overall stability and the open circuit voltage (*V*_oc_) [40–43]. Additionally, spacer cations integrated during the annealing process contribute to the film's flexibility, reducing defects and improving performance. The promising results of this approach have spurred significant interest in quasi-2D perovskites, which incorporate 2D structures into traditional 3D perovskites. These 2D perovskites are categorized into Ruddlesden-Popper (RP) and Dion-Jacobson (DJ) phases based on the nature of the spacer cation [44–46].

RP phases use monovalent cations, while DJ phases use divalent cations, leading to subtle differences in their crystal structures. The ideal Pb-I-Pb angle in the crystal structure is 180°, indicating no tilting. However, the flexible nature of RP phases, due to van der Waals (vdW) interactions between spacer cations, can potentially reduce structural distortion more effectively than the more rigid DJ phases. This flexibility can allow PSCs to have grains that grow more flatly and vertically, reducing grain boundary. Despite these advantages, the gap between the vdW interactions acts as a barrier that impedes charge-carrier transport. Additionally, the impact of these structural differences depends on whether the QW is thick or thin. Therefore, the exact impact of QW structural differences on performance remains under investigation [47–51].

Furthermore, additives and passivators are crucial for optimizing the crystal structure and enhancing both efficiency and stability of 2D perovskites. In 3D perovskites, methylammonium chloride (MACl) is commonly used as a metal halide additive to create intermediates that reduce oxidation [52]. However, there are numerous additive strategies for quasi-2D perovskite, including controlling Lewis basicity through solvent-assisted methods and precursor aggregation with additional spacer cations. A thorough understanding of the mechanisms behind Pb-I-Pb angles and effects of various passivators is essential for designing optimal quasi-2D perovskite compositions.

These optimization methods are expected to drive further advancements in perovskite device technology and facilitate the commercialization of PSCs [53–55]. Supporting this claim, comparisons of the compositions and performances of perovskite devices, particularly those based on the RP and DJ structures, as investigated by previous studies, provide valuable insights. Such comparisons allow for a comprehensive evaluation, and contribute to both commercialization and efficiency improvements in the field. In this review, scientific understanding and engineering perspectives of the quasi-2D perovskite is investigated, and the optimal structure quasi-2D and the device optimization is also discussed to provide the insight in the field.

## Three-Dimensional Perovskite (3D Perovskite)

Over the past few decades, extensive researches effort on PSCs utilizing 3D perovskite have led to notable improvements in PCE. The 3D perovskite, characterized by an octahedral ABX_3_ structure, can be engineered through precursor formulation. By combining an organic cation, a metal cation, and a halide anion in appropriate ratios, and using solvents like dimethyl sulfoxide (DMSO) to form a precursor, a thin film can be deposited onto a conductive substrate such as indium tin oxide (ITO)-coated glass via spin coating. Within the perovskite layer, the B-site cation is surrounded by six X-site anions, forming an octahedral structure.

**Fig. 1:**
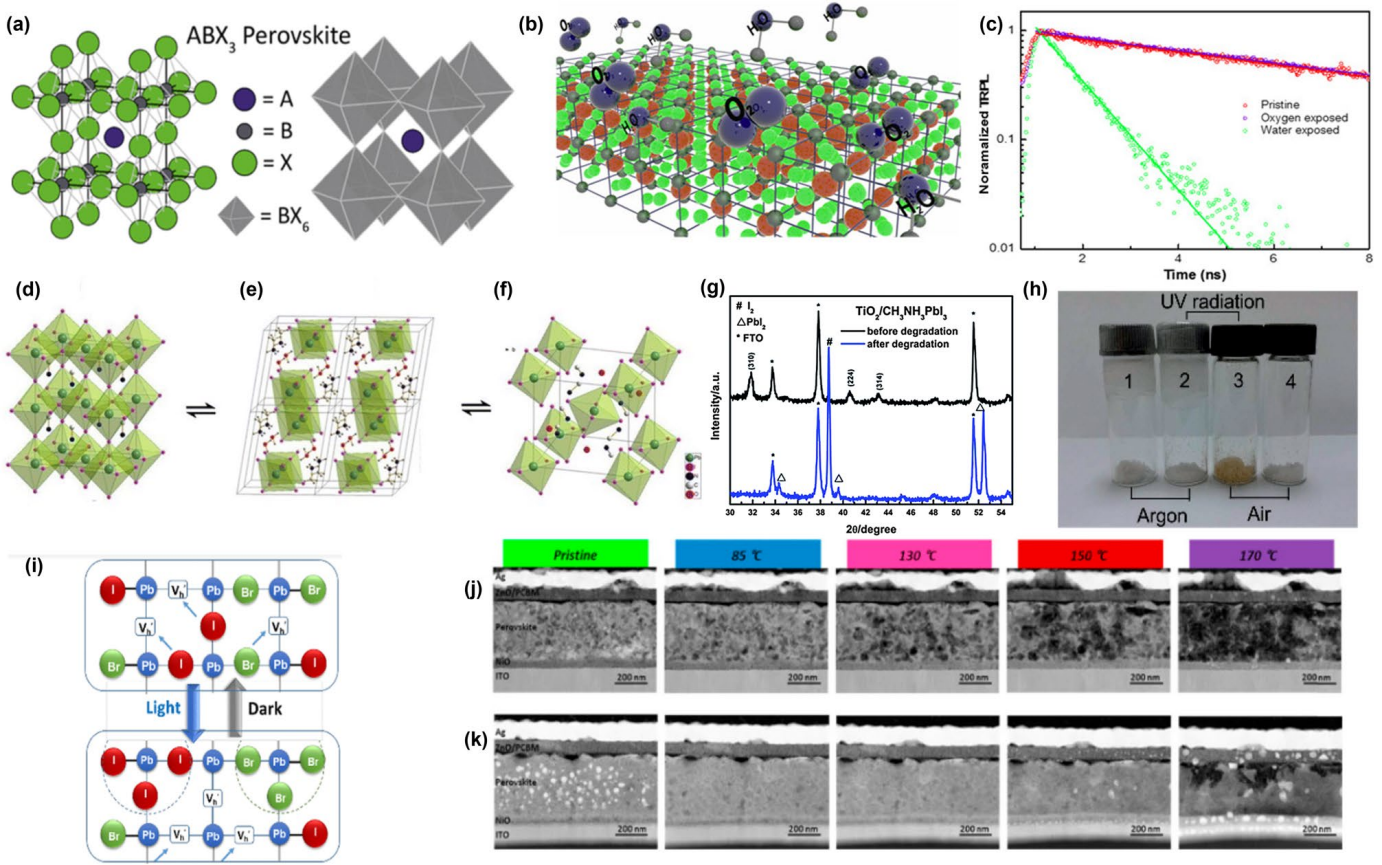
Negative factor of Three-dimensional (3D) Perovskite and it’s structure. **a** Basis of 3D perovskite structure. **a** is reprinted with permission from [56], copyright 2020 American Chemical Society. **b** Schematic Illustration about oxygen and water degradation and **c** indicated Photoluminescence decay kinetics of the CH_3_NH_3_PbI_3_ films before (red) and after exposure (oxygen: violet; water: green). **b** and **c** are reprinted with permission from [64], copyright 2018 American Chemical Society. **d** indicates the basic structure of Perovskite's MAPbI_3_, the cubic phase, **e** shows the structure of the monohydrate phase, CH_3_NH_3_PbI_3_·H_2_O, and **f** displays the structure of the dihydrate, (CH_3_NH_3_)_4_PbI_6_·2H_2_O. The position of the hydrogens on the (CH_3_NH_3_)^+^ ions and the water is not assigned in panels. **d-f** are reprinted with permission from [70], copyright 2015 American Chemical Society. **g** Using XRD, detect CH_3_NH_3_PbI_3_ film degradation according to **h** air and UV–vis stability. **g** and **h** are reprinted with permission from [75], copyright 2014 Journal of Materials Chemistry A. **i** Focused on halide ion migration in nanocrystals and nanostructured films, under the light irradiation. **i** is reprinted with permission from [80], copyright 2021 American Chemical Society. **j, k** shows scanning transmission electron microscopy (STEM) images which is heated in different step, **j** MA-, **k** Cs/FA/MA–PSC. **j** and **k** are reprinted with permission from, copyright 2020 Elsevier

Lead is the most commonly used material for the B-site cation, while iodine and bromine are frequently used for the X-site anion. This configuration results in a BX_6_ octahedral structure through corner-sharing interactions (Fig. 1a) [56]. The size of the A-site cation influences the regularity of the structure and particle size, with formamidinium (FA) and methylammonium (MA) being commonly used [57, 58]. The perovskite exhibits properties such as dielectricity, ferroelectricity, semiconductivity, superconductivity, and photovoltaic effects [59–63].

Recently, the demand for PSCs as photovoltaic devices has increased due to growing environmental concerns. Despite achieving remarkable PCEs over the past decades, a major challenge in commercializing PSCs is their low stability, which leads to device degradation. This degradation reduces efficiency and shortens device lifetime. Although significant efforts to address this issue, achieving long-term stability remains complex challenges. Before proposing methods to improve stability, it is crucial to discuss the key factors contributing to this problem.

### Moisture-Induced Degradation

Moisture stability is a first critical issue for perovskite materials (Fig. 1b). In ambient environments, exposure to airborne moisture or direct contact with water will cause the device to degrade. As shown in Fig. 1c, Bao et al*.* compared the time-resolved photoluminescence (TRPL) of CH_3_NH_3_PbI_3_ (MAPbI_3_) under water exposure compared to no exposure. The TRPL measurements revealed that a significant reduction in the PL lifetime of MAPbI_3_ films upon moisture exposure. Initially, the pristine perovskite film exhibits a PL lifetime of approximately 7.6 ns. However, after exposure to moisture, the PL lifetime drops dramatically to 0.87 ns. This experiment is noteworthy because the oxygen-exposed MAPbI_3_ films showed no significant changes, whereas the water-exposed films exhibited severe degradation, indicating that water exposure causes severe degradation of the perovskite films. This degradation can be interpreted as water makes the perovskite films more heterogeneous, increasing trap density and inducing excitons to undergo non-radiative recombination. Non-radiative recombination reduces the carrier lifetime, thereby shortening the device’s overall lifespan. To address these issues, accurate insights into the degradation mechanisms are required. To understand the mechanism in more detail, the stoichiometric Eqs. (1)–(5) representing the composition of the hydrate species are presented with structural illustrations (Fig. 1d–f) [64–71].

1 $${\text{CH}}_{{3}} {\text{NH}}_{{3}} {\text{PbI}}_{{3}} + {\text{H}}_{{2}} {\text{O}} \leftrightarrow {\text{ CH}}_{{3}} {\text{NH}}_{{3}} {\text{PbI}}_{{3}} \cdot {\text{H}}_{{2}} {\text{O}}$$

2 $${4}({\text{CH}}_{{3}} {\text{NH}}_{{3}} {\text{PbI}}_{{3}} \cdot {\text{H}}_{{2}} {\text{O}}) \leftrightarrow ({\text{CH}}_{{3}} {\text{NH}}_{{3}} )_{{4}} {\text{PbI}}_{{6}} \cdot {\text{2H}}_{{2}} {\text{O}} + {\text{3PbI}}_{{2}} + {\text{2H}}_{{2}} {\text{O}}$$

3 $$({\text{CH}}_{{3}} {\text{NH}}_{{3}} )_{{4}} {\text{PbI}}_{{6}} \cdot {\text{2H}}_{{2}} {\text{O}} \to {\text{4CH}}_{{3}} {\text{NH}}_{{3}} {\text{I}} + {\text{PbI}}_{{2}} + {\text{2H}}_{{2}} {\text{O}}$$

4 $${\text{CH}}_{{3}} {\text{NH}}_{{3}} {\text{PbI}}_{{3}} \leftrightarrow {\text{PbI}}_{{2}} + {\text{ CH}}_{{3}} {\text{NH}}_{{3}} {\text{I}} \leftrightarrow {\text{PbI}}_{{2}} + {\text{ CH}}_{{3}} {\text{NH}}_{{2}} + {\text{ HI}}$$

When MAPbI_3_ is exposed to ambient moisture, water molecules are absorbed into the perovskite lattice, forming a hydrated compound Eq. (1). This initial reaction introduces minimal structural disruption and results in the formation of a monohydrate phase (Fig. 1e). Notably, the monohydrate phase can revert back to the original perovskite structure upon drying, indicating the reversible nature of this initial hydration process. With prolonged moisture exposure, the MAPbI_3_ structure absorbs more water molecules, transitioning to the dihydrate phase as depicted in Eq. (2). This phase formation is accompanied by the production of lead iodide (PbI_2_), signifying partial degradation. PbI_2_ is an inorganic compound, usually yellow, which aligns with the color observed when perovskite degrades, forming as a primary degradation product. The dihydrate phase involves a structural reorganization from a three-dimensional network to a zero-dimensional framework of isolated PbI_6_^4−^ octahedra, stabilized by water molecules through hydrogen bonding (Fig. 1f). In the presence of excess liquid water, the dihydrate phase undergoes further irreversible decomposition. The reaction results in the formation of CH_3_NH_3_I (MAI) and PbI_2_, facilitated by the dissolution of CH_3_NH_3_^+^(MA) ions leading to the complete breakdown of the perovskite structure. Equation (4) outlines the overall degradation mechanism, highlighting MAI as an indicator of the initial stages of perovskite decomposition. Additionally, MAI further decomposes to CH_3_NH_2_ and HI, each as a volatile organic compound that catalyze the degradation process of perovskite materials. To prevent reaching the highly hydrated perovskite form where MAI is produced, it is crucial to avoid extensive moisture exposure. Therefore, employing moisture-blocking technique such as encapsulation is essential for maintaining perovskite stability and extending the lifespan of the devices [72–75].

### Oxygen-Induced Degradation

As mentioned in the previous part, water-exposed films are more significant problem than oxygen-exposed films. However, this does not imply that oxygen is harmless to perovskite films. As shown in Eq. (4), the initial stages of perovskite decomposition produce HI when exposed to water. It is suggested that perovskite exhibits a natural degradation behavior and that the HI formed acts as a catalyst in the degradation process, a phenomenon more pronounced in oxygen-exposed films. As shown in Eq. (5), perovskite degrades upon contact with moisture, forming CH_3_NH_3_PbI_3_⋅H_2_O, which subsequently breaks down into MAI and PbI_2_. This reaction leads to the formation of HI, as described in Eq. (6). Finally, when HI encounters oxygen in the air, it undergoes the oxidation–reduction reaction as shown in Eq. (7), resulting in the formation of 2I_2_ and 2H_2_O. HI, also known as hydroiodic acid, acts as a powerful reducing agent and reduces O_2_. This reaction produces I and water as byproducts, which can be observed as peaks in X-ray diffraction (XRD) studies conducted by Niu et al*.* The XRD spectra of the TiO_2_/MAPbI_3_ films before and after degradation show the disappearance of the original MAPbI_3_ peaks after degradation, indicating that MAPbI_3_ has transformed into different compounds. New peaks at 34.3°, 39.5°, and 52.4° correspond to the (102), (110), and (004) planes of the hexagonal 2H polytype PbI_2_. Additionally, a new peak at 38.7° is attributed to the (201) plane of orthorhombic I_2_. This suggests that exposure to air and moisture causes MAPbI_3_ to degrade, resulting in the formation of PbI_2_ and I_2_ as final products (Fig. 1g) [76].

5 $${\text{CH}}_{{3}} {\text{NH}}_{{3}} {\text{PbI}}_{{3}} \cdot {\text{H}}_{{2}} {\text{O }} \leftrightarrow {\text{ CH}}_{{3}} {\text{NH}}_{{3}} {\text{I }} + {\text{ PbI}}_{{2}}$$

6 $${\text{CH}}_{{3}} {\text{NH}}_{{3}} {\text{I }} \leftrightarrow {\text{ CH}}_{{3}} {\text{NH}}_{{2}} + {\text{ HI}}$$

7 $${\text{4HI }} + {\text{ O}}_{{2}} \leftrightarrow {\text{ 2I}}_{{2}} + {\text{ 2H}}_{{2}} {\text{O}}$$

### Light-Induced Degradation

Comparing the degradation of water-exposed and oxygen-exposed films reveals similar result. Therefore, it is essential to assess the degradation under light exposure using each film precursor. According to Le Chatelier's principle, a system at equilibrium will adjust to minimize the effect of changes in concentration, temperature, or pressure, thus making the degradation process more likely to proceed. Equations (4)-(6) are crucial for understanding the oxygen-exposed and light-exposed degradation process. Equation (6) is another degradation pathway for HI in light-exposed films, leading to the production of I_2_. The results of exposing both argon- and air-exposed precursors to ultraviolet (UV)-light are shown in Fig. 1h. As a result, the argon-conditioned precursor, which blocks the precursor reaction, does not react and remains unchanged, while the air-conditioned precursor turns brown color because of the presence of I_2_. I_2_ also negatively impacts the perovskite structure, contributing to structural instability, the formation of defect sites, and reduced photostability. These adverse effects facilitate I-ion migration (Fig. 1i) [77]. Consequently, addressing light-exposed perovskite degradation is critical. Under UV light, I in perovskite materials exhibits increased mobility, leading to halide ion migration, phase segregation, and the expulsion of molecular I_2_. These processes introduce defects and heterogeneities in the material, significantly accelerating degradation and negatively impacting the performance and stability of perovskite-based devices. Combining these results with the XRD measurements indicates that the end products of I_2_ and PbI_2_ form, detailing the degradation of the perovskite. It also shows that the initial degradation, initiated by MAI due to humidity, plays a significant role in the ongoing degradation process.

8 $${\text{2HI }} + hv \leftrightarrow {\text{ H}}_{{2}} + {\text{ I}}_{{2}}$$

### Heat-Induced Degradation

The organic cations in perovskites do not directly contribute to the electronic band structure. However, they significantly influence the structural, electronic, and optical properties of the hybrid halide materials by rotation with end of NH_3_^+^ through N^+^ – H···X hydrogen bonding between B, X. By manipulating the rotation mode, position, and orientation of the organic cations, the band gap, static dielectric constant, and absorption edges of the optical spectrum can be significantly tuned [78, 79]. Despite this, the lower thermal stability of these organic cations compared to other components, such as B and X, leads to thermal degradation at relatively low temperatures.

Seo et al*.* compared scanning transmission electron microscopy (STEM) images of MA-, Cs/FA/MA-PSCs to study degradation at different temperatures (Fig. 1j, k). These images reveal morphological changes as the temperature increases, emphasizing the effect of thermal stress on the material. At 85 °C, the perovskite film shows minimal change from its initial state, with a relatively smooth surface and well-defined grain boundaries without an obvious sign of void formation or particle aggregation, indicating stability under moderate thermal burden. Upon heating to 130 °C, early signs of thermal degradation appear, with small voids and minor surface roughness, partial decomposition and the loss of volatile components. Grain boundaries are still visible, but there is evidence of some structural weakening. At 150 °C, decomposition accelerates, with larger voids and small particles scattered across the surface, signifying increased material loss and structural breakdown. When the temperature reaches 170 °C, the film suffers severe degradation, with extensive voids and large grain aggregations. The structural integrity of the perovskite is greatly compromised, making the film appear highly irregular and fragmented. This level of degradation indicates significant volatilization and phase separation of the organic components, resulting in a complete breakdown of the perovskite structure.

The observed morphological changes can be attributed to several main degradation mechanisms: volatilization of organic components, phase separation, and ion migration. As temperature increases, the organic components such as MA, and Cs/FA/MA volatilize, leading to formation of voids and structural collapse. Phase separation occurs in mixed halide perovskites at high temperature, creating regions with different compositions and disrupting film uniformity. High temperatures also promote ion migration, leading to the defect accumulation and crystal lattice disruption, and changing rotation mode, further contributing to the morphological changes. These structural changes significantly impact on the performance of PSCs. The formation of voids and phase separation create barriers to charge carrier transport, increasing recombination rates and reducing overall device efficiency. Loss of uniformity in the perovskite film reduces light absorption and increases instability under continuous illumination. The observed degradation at relatively moderate temperatures (150–170 °C) suggests the importance of thermal management for the long-term operational stability of PSCs in ambient applications. STEM imaging provides valuable insights into the thermal degradation of perovskite films [80].

The observed degradation mechanisms, including volatilization of organic components, phase separation, and ion migration, highlight the importance of improving the stability of perovskite materials. Additionally, as perovskite nuclei form within the liquid precursor and transition into a solid crystal phase, any unevenness in this phase can lead to lattice mismatch due to mechanical properties such as strain [81–85]. It is evident that the degradation of 3D perovskite is influenced by various environmental factors such as water, oxygen, light, and heat. Furthermore, the degradation of optoelectronic performance due to mechanical properties cannot be ignored. Addressing these issues is essential for developing durable and efficient perovskite solar cells for commercial applications. Therefore, further investigation and mitigation strategies are necessary to address the issues mentioned in this section.

## Two-Dimensional Perovskite (2D Perovskite) & Quasi-2D Perovskite

2D perovskites were first investigated in 1990s [86]. The structure of 2D perovskite composed A'_(2or1)_A_(n-1)_B_(n)_X_(3n+1)_ structure, which includes a large organic ammonium cation(A') as a spacer. The introduction of a large spacer cation into a 3D perovskite disrupts its three-dimensional network, can caused low PCE. As the spacer cations are inserted between the corner-sharing BX_6_ octahedra, they break their connectivity [87, 88]. However, this process creates a 2D perovskite, where the spacer cations serve as barriers, increasing exciton binding energy, reducing non-radiative recombination, and enhancing stability due to longer carrier lifetimes [89, 90].

**Fig. 2:**
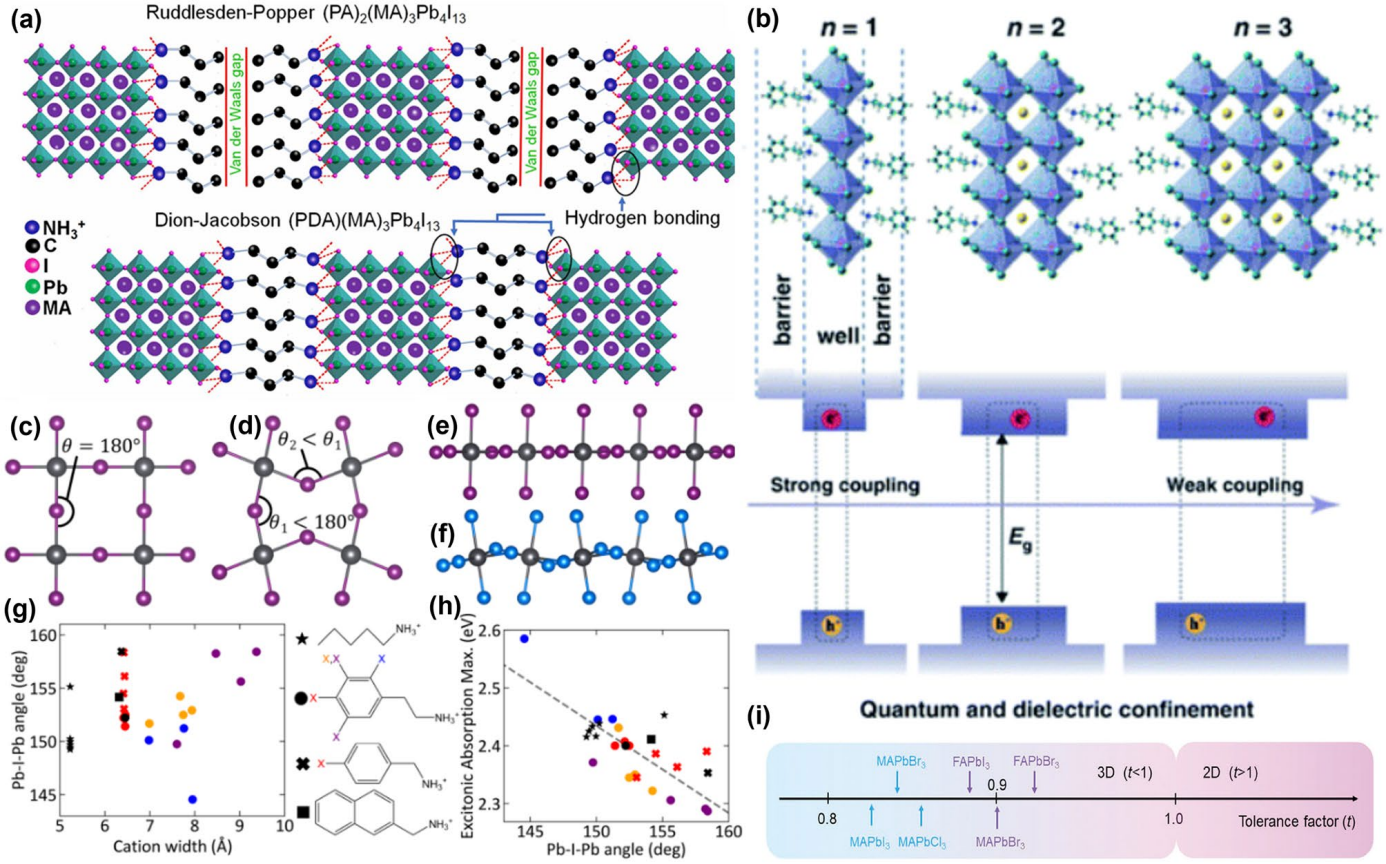
Structure of Two-dimensional (2D) perovskite, using various organic cation can adjust structure angle to enhance stability and performance. **a** Schematic Illustration of RP and DJ phase 2D layered perovskites. **a** is reprinted with permission from [91], copyright 2019 Joule. **b** Structure of 2D perovskite that has quantum-well, barrier, exciton binding energy and bandgap which is can change so many different type for different n ratio. **b** is reprinted with permission from [107], copyright 2021 Light: Science & Applications. **c** Structure of perfect inorganic 2D perovskite cubic with angle θ = 180°. **d** Structure of distorted inorganic 2D perovskite cubic with angle θ2 < θ1 < 180°. **e** Planar and** f** corrugated inorganic frameworks. **g** Geometry, i.e., the length and width, and the composition, that drives chemical interactions within the organic and between the organic and inorganic layers. And it causes to different **h** excitonic absorption Max. **c**–**h** are reprinted with permission from [115], copyright 2022 Annual Review of Physical Chemistry. **i** for each different structure has different tolerance factor (t), (t) > 1 is 2D like, (t) < 1 is 3D like. **i** is reprinted with permission from [128], copyright 2022, Wiley–VCH

Understanding the mechanism requires detailed investigation of the 2D perovskite structure. 2D perovskite has two types of structures. There are two main types of 2D perovskite structures, categorized by the type of A', which is a spacer cation with an ammonium group attached to the ligand. When the ammonium spacer is a monovalent cation, it forms Ruddlesden-Popper (RP) perovskites, and when it is a divalent cation, it forms Dion-Jacobson (DJ) perovskites (Fig. 2a) [91]. The ammonium groups form strong hydrogen bonds with the typical BX_6_ octahedral structure of perovskites, resulting in strong binding. In this configuration, the organic spacer cations attach to both sides of the perovskites structure, acting as a barrier within the perovskite. These barriers have relatively insulating properties that limit charge transport, but at the same time, show hydrophobic properties that protect the perovskite from external contact, similar to encapsulation. This explains why 2D perovskites are highly stable in ambient environments and exhibit quantum confinement effect [38].

### Quantum Confinement

Quantum confinement occurs when the size of a semiconductor, such as perovskite crystal is reduced to below the exciton Bohr radius, resulting in discrete energy levels for excitons (electron–hole pairs). This confinement significantly alters the electronic properties of the material, leading to an increased bandgap due to energy level quantization. Additionally, it enhances Coulomb interactions between electrons and holes, making excitonic effects more pronounced. In low-dimensional perovskite structures, such as quantum wells, nanowires, and quantum dots, quantum confinement results in size-dependent optical and electronic properties that differ from those of bulk materials. These properties include more defined absorption and emission features, which are advantageous for optoelectronic applications. Consequently, excitons remain bound and do not dissociate, demonstrating that the organic cation spacer acts as a barrier to charge transport [92–96].

Back to the 2D perovskite’s structure, perovskite has barriers on both sides, effectively isolating the excitons within their own “well”. This configuration is analogous to a well containing water, where the well represents the interface between substances with different dielectric constants. The term "wells" refers to this isolated perovskite structure, with the behavior of excitons influenced by the number of these wells. The n-value in the perovskite precursor specifies the number of wells, or perovskite layers. As the n-value approaches infinity, exciton binding energy decreases, allowing freer charge transport and resulting in a reduced bandgap which is characteristic of standard 3D perovskites. An increasing n-value thus leads to a reduction in the barrier, diminishing stability, increasing non-radiative recombination, and affecting efficiency. Conversely, lower n-values emphasize the characteristics of 2D perovskites. However, if the n-value is too low, the exciton binding energy becomes excessively high, preventing necessary exciton dissociation for effective charge transport. Researchers suggest that an ideal n-value is between 1 and 5, though the theoretical basis for this range is not yet fully understood and requires further investigation [97, 98]. This arrangement of semiconducting perovskite wells and insulating A' barriers results in a dielectric confinement effect [99–103].

### Dielectric Confinement

Dielectric confinement occurs when the dielectric constant of the surrounding medium differs from that of the material, influencing the Coulombic interactions within the material. This difference increases exciton binding energy by enhancing the Coulombic attraction between electrons and holes. Additionally, the lower dielectric constant of the surrounding environment reduces the screening of these interactions, making their effects more significant. In perovskites, dielectric confinement leads to higher exciton binding energies and reduces non-radiative recombination losses. Stronger Coulomb interactions prevent energy loss through defects and grain boundaries, thereby enhancing the stability and efficiency of optoelectronic devices, such as solar cells and LEDs, where maintaining high exciton binding energy is key for optimal performance [104–106].

These quantum- and dielectric-confinements create a well structure with a surrounding barrier, resulting in a quantum well configuration (Fig. 2b) [107]. Both RP and DJ exhibit such structures, though they show slightly different structures depending on the number of functional groups in the spacer cation A'. This will be discussed further in the sections on RP and DJ. As previously explained, the 2D perovskite structure inhibits the dissociation of excitons and block external contact with the perovskite. As a result, excitons accumulate within the perovskite, increasing the charge potential. High exciton binding energy minimizes non-radiative recombination, where energy is lost as heat rather than light due to defects or grain boundaries. Additionally, the hydrophobic nature of the A' cation enhances stability [108].

### Structure Factor

Comparing 2D and 3D perovskites made using the quantum well structure, the key difference is that 2D perovskites have bonds that act as a barrier between A' and the perovskite. In 2D perovskites, the A' group, an ammonium functional cation, plays a crucial role. While the chemical properties of this cation have been addressed in the context of the quantum well structure, we now focus on its physical impact on perovskite properties. The perovskite ideally maintains a consistent Pb-I-Pb angle and lattice alignment, which enable perfect vertical growth and optimal cell performance. However, achieving this ideal condition is challenging. The physical characteristics of the A' cation influence the Pb-I-Pb angle and the Pb-I bond length within the perovskite lattice [109–111]. Perovskites typically form by stacking BX_6_ octahedral lattices in layers. However, research shows that this octahedral structure is not always uniformly formed [112]. For instance, if the Pb-I-Pb angle were consistently 180°, the perovskite would achieve an ideal structure with maximum efficiency of 33.7%, as predicted by Shockley-Queisser (SQ) theory [113]. However, lattice distortion induced by the A' cation in 2D perovskites can break the symmetry and affect the efficiency of the device [114]. The A' cation’s characteristics—such as ligand length, aromatic rings, and the number of ammonium groups—can induce significant lattice distortion. As mentioned earlier, if the Pb-I-Pb angle becomes ideal θ (θ = 180°), an ideal symmetric perovskites structure with no tilting will be formed (Fig. 2c). However, if the angle is less than θ (θ_2_ < θ_1_ < 180°), perovskite lattice distortion will occur, and this smaller Pb-I-Pb angle tends to increase the bandgap, due to shorter bond length (Fig. 2d). The bond angles also affect the stacking of the inorganic framework: the set of θ angles forms Fig. 2e, and the set of θ_1_, θ_2_ forms Fig. 2f.

The impact of the A' cation on the Pb-I-Pb angle is linked to the cation's width. In Fig. 2g, a scatter plot shows the relationship between cation width and the Pb-I-Pb bond angle for various experimental 2D perovskites. For instance, basic phenylethylammonium (PEA) cations (represented by black circles) cause minimal distortion, whereas PEA cations with 2-position substituents (represented by blue circles) lead to larger distortion and a more corrugated inorganic framework. Figure 2h illustrates a strong correlation between the Pb-I-Pb bond angle and excitonic absorption maximum [115–123]. The grey line indicates that as the Pb-I-Pb angle decreases (signifying increased distortion), the excitonic absorption energy increases (blue shift). This relationship highlights how cation-induced structural changes affect the optical properties of 2D perovskites. Knutson et al. found that increased Pb-I-Pb angles decrease orbital overlap, lowering the valence band energy, while structural distortions enhance anti-bonding interactions, raising conduction band energy and widening the bandgap [124, 125].

In conclusion, the specific characteristics of A' cations, including their geometry and chemical composition, play a crucial role in determining the structural and also optical properties of 2D perovskites. The Pb-I-Pb bond angle serves as a key indicator of lattice distortion, which directly impacts the material's bandgap and efficiency. Further research into these relationships will provide deeper insights into optimizing perovskite structures for enhanced performance.

### Crystal Plane Growth

It has been discovered that the A' cation significantly influences the perovskite lattice structure, causing lattice distortion that affects optoelectronic applications such as bandgap and photoluminescence (PL). To understand these properties in detail, the Goldschmidt Tolerance Factor (τ) is introduced. This factor, which relates to the quantum confinement effect experienced by excitons within the perovskite lattice, is calculated using the formula:

9 $$\tau = \frac{{r_{{\text{A}}} + r_{{\text{X}}} }}{{\sqrt 2 \left( {r_{{\text{B}}} + r_{{\text{X}}} } \right)}}$$

where *r*_A_, *r*_B_, and *r*_X_ represent the ionic radii of the A-site cation, B-site cation (typically Pb in lead halide perovskites), and the halide anion, respectively [126]. The tolerance factor provides insight into how well the ions fit into the perovskite crystal structure [127].

Typically, a τ ranging from 0.8 to 1.0 indicates a stable cubic or slightly distorted 3D perovskite structure. If τ is less than 0.8, the structure tends to be excessively distorted, leading to less stable or non-perovskite phases. Conversely, τ values exceeding 1.0 imply lattice strain and often result in the formation of non-perovskite phases or highly distorted structures. For 3D perovskites, achieving a tolerance factor within the ideal range (0.8 to 1.0) is crucial for maintaining structural stability. Common A-site cations like MA, FA, or Cs are used because their ionic radii help to achieve a stable tolerance factor. In contrast, 2D perovskites have a more relaxed tolerance factor requirement due to their layered structure. The large organic spacer cations can accommodate a broader range of tolerance factors (over 1.0), allowing for greater lattice distortions while maintaining stability (Fig. 2i) [128]. This flexibility provided by the relaxed tolerance factor in 2D perovskites results in enhanced stability against environmental factors like moisture and thermal stress, because the large organic cations buffer internal strain and absorb structural distortions. Additionally, the organic spacer layers isolate the inorganic perovskite layers, reducing the impact of lattice mismatches and further enhancing the material’s overall stability [129].

2D perovskites are assembled from various lattices influenced by different Pb-I-Pb angles due to the A' cations. These perovskites exhibit an average tolerance factor (τ) and do not strictly conform to either 2D or 3D configurations alone. The average number of perovskite lattices, defined by the n-value, leads to the classification of quasi-2D perovskites. While, "2D perovskite" can be used for structural categorization, "quasi-2D" is more appropriate for describing components in completed devices [130]. Quasi-2D perovskites are typically fabricated as thin films through solution process and their crystal plane states can be analyzed using XRD and grazing-incidence wide angle X-ray scattering (GIWAXS) techniques.

**Fig. 3:**
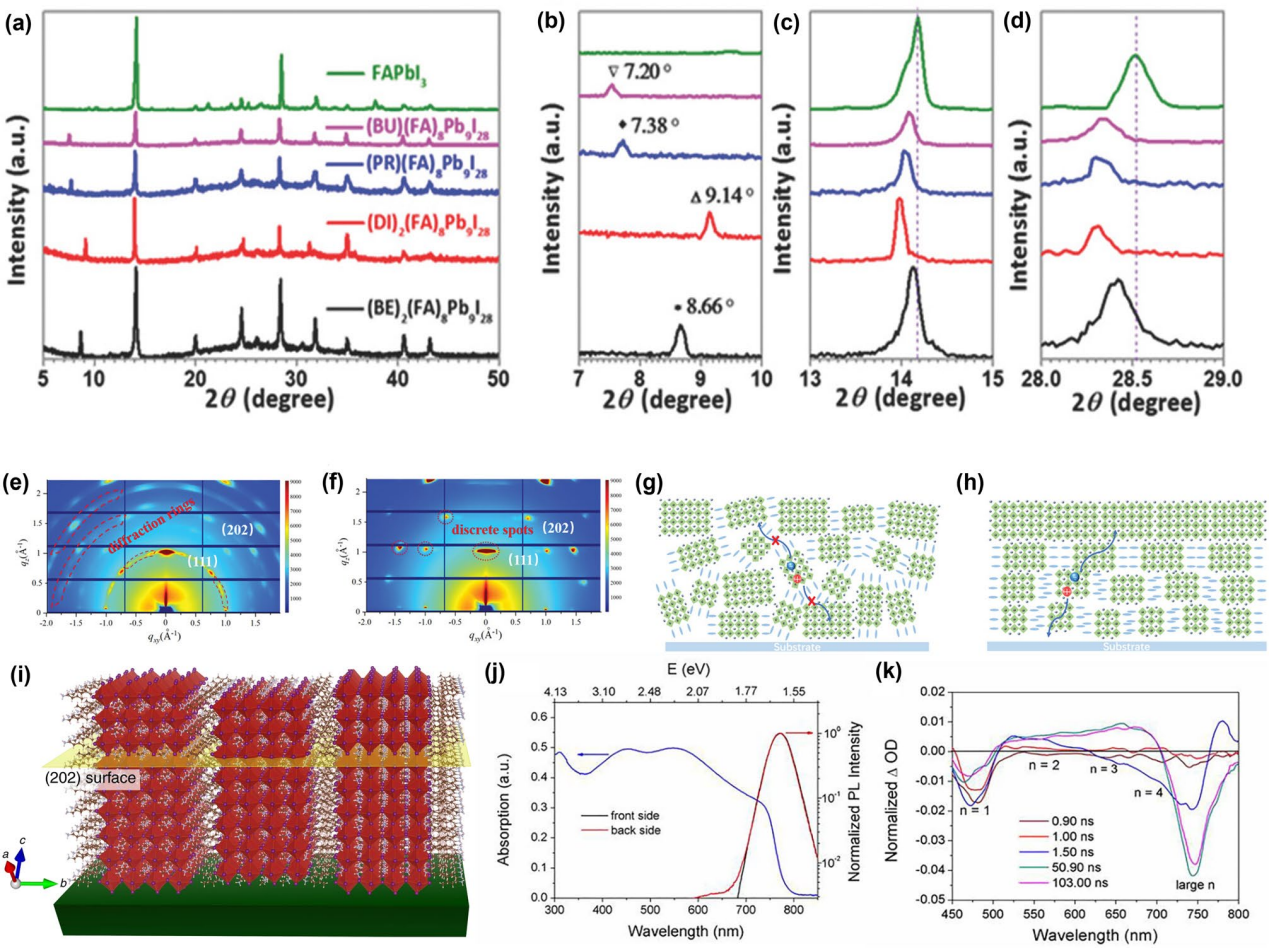
Ideal crystal plane growth of 2D perovskite: Vertical orientation. **a** XRD peak of each different 2D perovskite: **b** each different organic cation can be shifted to 8.66°, 9.14°, 7.38°, and 7.20° **c** 14° and **d** 28° can be reached perfect crystal plane. **a**–**d** are reprinted with permission from [131], copyright 2018 Advanced Energy Materials. **e, f** GIWAX images of 2D (BA)_2_MA_4_Pb_5_I_16_ perovskite film without **a** and with **b** DMSO. **g, h** Charge-transfer diagram of 2D (BA)_2_MA_4_Pb_5_I_16_ perovskite film without **c** and with **d**, respectively. **e**–**h** are reprinted with permission from [132], copyright 2023 Advanced Electronic Materials. **i** Illustration of an orthorhombic (101) vertically oriented 2D perovskite structure, with (202) planes parallel to the substrate. **i** is reprinted with permission from [133], copyright 2018 Nature communication. **j** Top and bottom, **k** small and large *n* values in quasi-2D perovskite are revealed PL spectra. **j** and **k** are reprinted with permission from [137], copyright 2022 Elsevier

Zheng et al*.* used XRD to analyze films with different A' cations, revealing consistent peaks for each perovskite film. Four selected ammonium salts (C_6_H_5_CH_2_NH_3_I (BEI), (CH_3_)_2_NH_2_I (DII), IH_3_N(CH_2_)_3_NH_3_I (PRI_2_), and IH_3_N(CH_2_)_4_NH_3_I (BUI_2_)) were used to fabricate the device. The XRD patterns of quasi-2D perovskites and FAPbI_3_ thin films, shown in Fig. 3a–d, exhibit strong peaks at approximately 14° and 28° which are characteristics of the perovskite phase, with no PbI_2_ peaks observed, indicating a preferential orientation of crystallites (Fig. 3a). Notably, the peaks for (BE)_2_(FA)_8_Pb_9_I_28_ and (DI)_2_(FA)_8_Pb_9_I_28_ are very intense, suggesting that these perovskites have highly oriented crystals. In contrast, the peaks for (PR)(FA)_8_Pb_9_I_28_ and (BU)(FA)_8_Pb_9_I_28_ films are weaker, indicating poorer crystallinity. All four quasi-2D perovskite films exhibit new diffraction peaks at around 8.66°, 9.14°, 7.38°, and 7.20°, corresponding to the 2D perovskite structure (Fig. 3b). Additionally, Fig. 3c and d reveal slight shifts in peaks around 14° and 28° toward smaller angles, consistent with PL spectra shifts, indicating changes in crystal lattice constants due to the incorporation of different ammonium salts, which alter the c-axis of the perovskite unit cell mentioned in the previous section [131].

To further investigate the crystal orientation in 2D (BA)_2_MA_*n*–1_Pb_*n*_I_3*n*+1_ perovskites, Pan et al*.* performed GIWAXS measurements. The peaks corresponding to the (111) and (202) planes at q_zy_ = 1.0 and 2.0 Å^−1^ respectively, align with the XRD peaks at 14.1° and 28.6°. With the absence of the DMSO additive, the 2D perovskite film showed random crystal orientation, with pronounced diffraction rings and weak diffraction spots (Fig. 3e). The DMSO-modified film, however, exhibited strong, sharp, and discrete Bragg spots for the (111) and (202) planes, rather than diffraction rings, suggesting enhanced vertical crystal orientation (Fig. 3f). This enhancement suggests that DMSO manipulation improved vertical charge transport by creating a more ordered structure, as depicted in Fig. 3g. However, after DMSO manipulation, the less oriented 2D layered structure transforms into a highly oriented structure, which significantly improves charge transport. The optimized vertical orientation results in a direct path for charge transport and reduced lateral transport (Fig. 3h) [132].

GIWAXS results reveal the strong vertical orientation of BA_2_MA_3_Pb_4_I_13_ in 2D perovskite films as illustrated in Fig. 3i. Evidences by the sharp XRD peaks and distinct GIWAXS Bragg spots suggest an optimal vertical alignment [133]. This ideal vertical orientation is achieved through processes such as controlled evaporation of the solution, prevention of homogeneous nucleation, and precise adjustment of the A' cation. Understanding how these structures form during spin-coating and annealing is crucial. During the spin-coating process, homogeneous nucleation within the bulk of the solution typically leads to the growth of randomly oriented crystals due to the isotropic environment. In some cases, crystals resulting from homogeneous nucleation can achieve preferential orientation when deposited on a substrate, particularly if the crystals have anisotropic dimensions and an orientation that maximizes interactions with the substrate, such as vdW attractions. However, this scenario generally promotes horizontal, rather than vertical, orientation of the 2D crystal plates to maximize vdW interactions. The strong vertical orientation observed indicates that suppression of homogeneous nucleation and dominance of heterogeneous nucleation at interfaces. Identifying which interface—substrate-liquid, liquid–air, or both—facilitates this heterogeneous nucleation is key to optimizing vertical orientation in 2D perovskite thin films for high-performance devices [134–136].

Based on this understanding, quasi-2D perovskites are developed with unique optoelectronic properties according to their average n-values. Lower n-values exhibit characteristics more akin to 2D perovskites, with high exciton binding energies and stability. Conversely, higher n-values resemble 3D perovskite characteristics, with enhanced charge transport due to reduced barriers and lower exciton binding energy, but without fully exhibiting well effects. Figure 3j, k illustrates these optoelectronic properties. Figure 3j shows the absorption peaks for 3FBAI-based quasi-2D perovskites, with mixed n-values of 2 and 1 showing peaks at 547 and 453 nm, respectively, and a peak at 740 nm corresponding to the 3D phase (n = ∞). Lower n-values absorb shorter wavelengths, appearing blue-shifted and exhibiting multiple peaks., evidencing the average nature of n-values in quasi perovskites [137]. Additionally, by dividing into the back side and front side, it can be observed that the lower n-values occupy the bottom part of the cell, while the higher n-values occupy the top part of the cell. Furthermore, Fig. 3k shows the results from femtosecond transient absorption (TA) measurements. A pump pulse (325 nm, 1 kHz, 100 fs) was used to excite the perovskite film, and the changes in absorption (△*A*) were recorded as functions of both time and wavelength. The TA spectra of quasi-2D films display several photobleaching peaks between 450 and 710 nm, corresponding to the small n phases of quasi-2D perovskites due to charge carrier filling upon excitation. The peak at 740 nm represents the 3D phase (n = ∞). These photobleaching peaks align with those observed in the steady-state absorption spectrum. TA spectra of quasi-2D films closely resemble those of low-dimensional 2D perovskite films, except for the absence of the 740 nm peak in the 2D films. Therefore, TA measurements confirm the formation of multiple phases in quasi-2D films [138–140].

These observations confirm that the characteristics of quasi-2D perovskites are influenced by various physical and chemical properties. The next section will investigate deeper fundamental aspects of the A' cation, including how its type, shape, and symmetry affect the properties of quasi-2D perovskites.

## Ruddlesden-Popper (RP) Perovskites

**Fig. 4:**
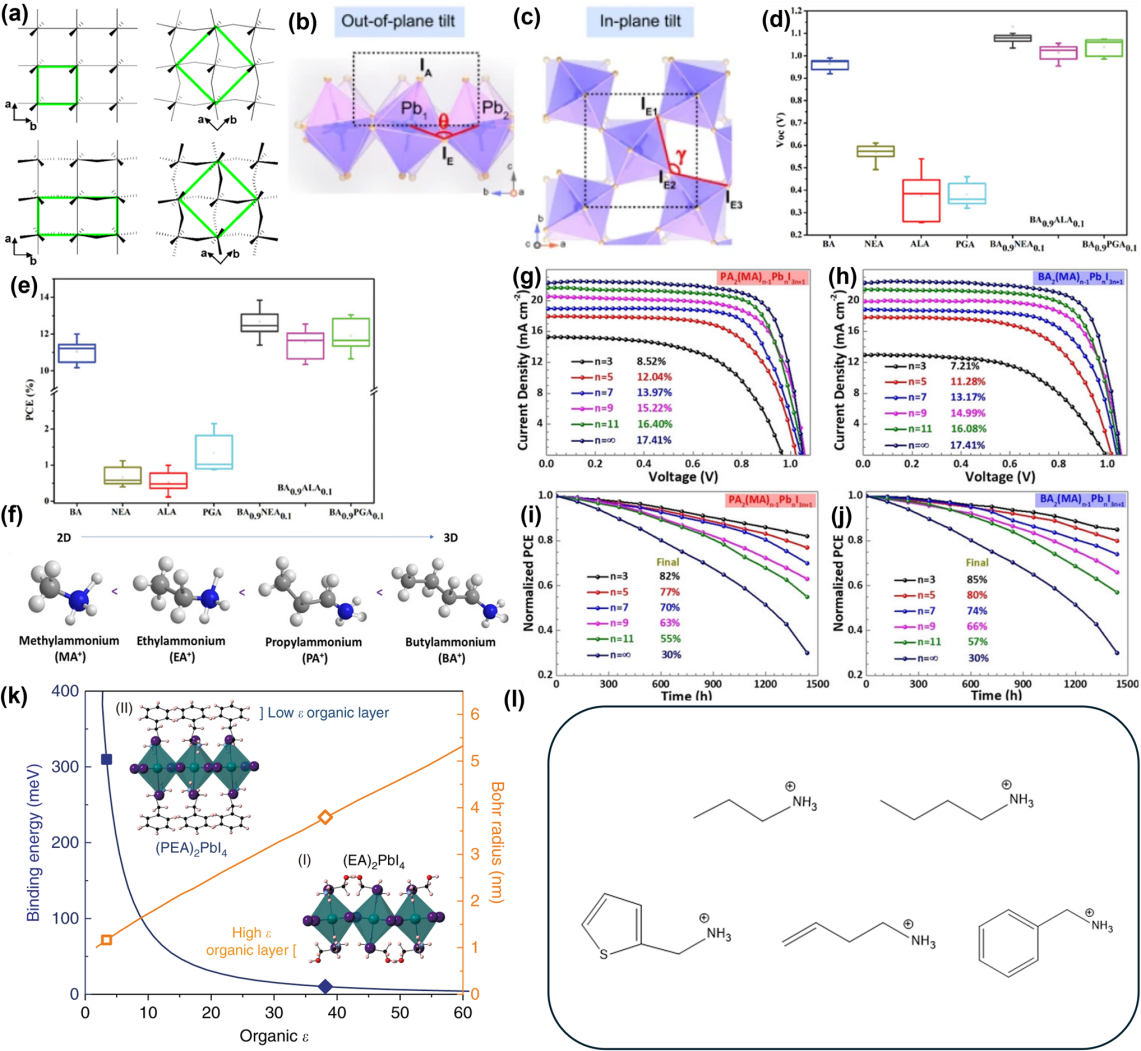
Ruddlesden-Popper perovskite (RP perovskite) distortion affected organic cation in many parts of means. **a** Undistorted lattice, in-plane distortion, out-of-plane distortion, and combined in- and out-of plane distortion. The green box highlights the unit cell that describes each lattice. **a** is reprinted with permission from [124], copyright 2005 American Chemical Society. **b** Out-of-plane tilt, **c** in-plane tilt are described in detail by Schematic Illustration. **b** and **c** are reprinted with permission from [141], copyright 2022 Nature communication. **d** The characterization of NEA, ALA, PGA, BA_0.9_NEA_0.1_, BA_0.9_ALA_0.1_, BA_0.9_PGA_0.1_. *V*_oc_ and **(e)** PCE statistic distribution. **d** and **e** are reprinted with permission from [151], copyright 2019 Advanced Energy Materials. **f** Schematic Illustration of each different organic cation length electronical behavior. **g**, **h** *J*–*V* curves and **i, j** normalized PCE variation curves of PA_2_(MA)_*n*−1_Pb_*n*_I_3*n*+1_, and BA_2_(MA)_*n*−1_Pb_*n*_I_3*n*+1_ (*n* = 3, 5, 7, 9, 11, and ∞) perovskite devices. **f**–**j** are reprinted with permission from [156], copyright 2019 ChemSusChem. **k** Exciton-binding energy and Bohr radius. The exciton-binding energy and Bohr radius as a function of organic-group dielectric constant in 2D perovskites as predicted by the image charge model. Calculated exciton-binding energies and Bohr radii of PEA (square) and EA (diamond) based 2D perovskites are indicated. Inset (I): Lattice structures of (HOCH_2_CH_2_NH_3_)_2_PbI_4_ (2D_EA perovskites) and (II): (C_6_H_5_(CH_2_)_2_NH_3_)_2_PbI_4_ (2D_PEA perovskites). **k** is reprinted with permission from [157], copyright 2019 Communications Physics. **l** Shape of each monovalent organic cation PA(Propylammonium), BA(Butylammonium), ThMA(2-thiophenemethlyammonium), BEA(1-amino-3-butene), PEA(Phenylethylammonium)

Quasi-2D perovskites form a BX_6_ lattice through the coordination of metal cations and halide anions. This lattice is illustrated in Fig. 4a, which depicts various distortions: undistorted lattice, in-plane distortion, out-of-plane distortion, and a combination of both [124]. The ideal 180° angle tilts within the *a, b* plane, influencing the perovskite's bandgap and optoelectronic properties. These tilts are categorized into in-plane and out-of-plane tilts, relating to 2D and 3D planes, respectively. Figure 4b, c illustrates these out-of-plane and in-plane tilts, where out-of-plane tilt indicates the deviation of the Pb-I-Pb angle in 3D structure. In contract, in-plane tilt, considering the I-I-I angle with 90° being ideal, is viewed from above within the *a, b* plane, ignoring the c-axis, thus representing a 2D perspective. This mechanism is consistent in both DJ and RP perovskites, providing detailed insights into how tilt influences their properties [141]. Unlike typical 3D perovskites, where the angle varies based on the organic cation (e.g., MA, FA), in 2D perovskites, the larger organic cation A' significantly affects the lattice structure. RP perovskites, which incorporate monovalent A' cations have a distinct arrangement where the A' cation is positioned on the outer halide anion side of the crystal as shown in Fig. 2a. Consequently, the alkyl ligands not attached to the ammonium on the A' cation form a vdW barrier between perovskite layers, which introduces structural flexibility. The presence of vdW forces allows the alkyl ligand to move freely, as it is not tightly bound to the perovskite [142–144]. Moreover, these forces also prevent the ligands from moving arbitrarily, thereby mitigating severe tilting and providing a corrective effect. This flexibility allows RP perovskites to alleviate tilt more effectively and exhibit better PCE compared to DJ perovskites, making them easier to handle. In this section, we will focus on the characteristics of RP perovskites.

RP perovskites, with the general formula A′_2_A_(n-1)_B_(n)_X_(3n+1)_, exhibit more flexible characteristics compared to DJ perovskites. However, this flexibility also increases lattice spacing, potentially negatively impacting charge transport. Moreover, longer or more rigid ligands may adversely affect efficiency. Fortunately, the presence of vdW barriers in RP perovskites allows for mitigation of these issues due to their excellent flexibility [144]. To explore these aspects in greater detail, refer to the study by Zhou et al. Zhou et al. explored these aspects by comparing the PCE of RP perovskites with linear cations versus those with phenyl ring A′ cations, highlighting significant differences in optoelectronic properties. In this experiment, MA series perovskites with linear butylammonium (BA) cations demonstrated a *V*_*oc*_ of 0.988 V and a PCE of 11.71% (Fig. 4d, e). Compared to perovskites solely composed of phenyl ring A′ cations, such as 1-naphthalene ethylammonium (NEA), allylammonium (ALA), and propargylammonium (PGA) resulted in significantly lower PCE around 2%, with *V*_*oc*_ below 0.6 V. The reduced efficiency in phenyl ring perovskites is attributed to their increased rigidity, which leads to greater lattice distortion and less optimal charge transport. Such bulky and rigid cations also provide further insights into the tilting that occurs as perovskite lattices form a networking structure. The large volume of spacer cations exacerbates this tilting, leading to significant lattice distortion. Therefore, forming A′ cations solely from phenyl cations may be impractical.

Conversely, the efficiency of perovskites with a small addition of phenyl ring A′ has been observed to increase. This effect can be attributed to the π–π stacking interactions facilitated by phenyl rings [146, 147]. This efficiency increases results from Jahn–Teller distortions caused by the different structures of linear and phenyl rings, where steric hindrance causes the π interaction orbitals of the benzene ring to overlap with the orbitals of surrounding atoms, stabilizing the donor and acceptor [148, 149]. The stabilized donor and acceptor not only reduce voltage loss due to non-radiative recombination but also facilitate charge transfer [150]. This resulted in increased *V*_*oc*_ of 1.09, 1.03, and 1.06 V for (BA_0.9_NEA_0.1_)_2_MA_3_Pb_4_I_13_, (BA_0.9_ALA_0.1_)_2_MA_3_Pb_4_I_13_, and (BA_0.9_PGA_0.1_)_2_MA_3_Pb_4_I_13_ devices respectively, which are higher compared to 0.97 V in BA_2_MA_3_Pb_4_I_13_—based devices. The efficiency of the PCE also shows a similar trend, adding credibility to the research. This study provides solid evidence that while the A′ cation does not change the direct bandgap, the lattice distortions it induces are impactful [151–154]. To understand the stability behavior of 2D cations, stability study using NEA, ALA have been summarized in Table 1 for reference [48, 155].

**Table 1:** PCE of Quasi-2D perovskite solar cells

Perovskite	A′	PCE	N (Number of Layer)	Year	Device structure	Perovskite Structure	Stability	Refs
Ruddlesden-Popper Perovskite	PA	17.23%	-	2019	FTO/mTiO_2_/perovskite/Spiro-OMeTAD/Au	PA_2*x*_FA_0.79_MA_0.16_Cs_0.05_Pb_1+*x*_I_2.5+4*x*_Br_0.5_	PCE of around 50% retained under average relative humidity, ambient atmosphere over a period of 2000 h	[158]
	BA	12.81%	N = 2, 3, 4	2018	ITO/PEDOT:PSS/perovskite/PCBM/BCP/Ag	(BA)_2_(MA_0.8_FA_0.2_)_3_Pb_4_I_13_	PCE of around 88% retained under ambient atmosphere, 40% ~ 60% relative humidity over a period of 1300 h	[159]
		13.92%	N = 4	2019	FTO/NiO_*x*_(with Cu, Cs)/perovskite/PCBM/Ag	BA_2_MA_3_Pb_4_I_13_	PCE of around 80% retained under N_2_ atmosphere, 35% ~ 40% relative humidity and 85 °C over a period of 250 h	[160]
		15.71%	N = 5	2021	ITO/SnO_2_/perovskite/Spiro-OMeTAD/Ag	BA_2_MA_4_Pb_5_I_16_With Ti_3_C_2_T_*x*_	PCE of around 80% retained under ambient atmosphere, 55 ± 5% relative humidity over a period of 750 h	[161]
		17.26%	N = 4	2019	ITO/PTAA/perovskite/C_60_/BCP/Ag	BA_2_MA_3_Pb_4_I_13_	PCE of around 96% retained under N2 atmosphere over a period of 2000 h	[162]
		18.04%	N = 5	2020	ITO/PTAA/perovskite/C_60_/BCP/Ag	BA_2_(MA_0.8_FA_0.15_Cs_0.05_)_4_Pb_5_I_16_	PCE of around 85% retained under N_2_ atmosphere, 60 °C over a period of 500 h	[163]
	ThMA	15.42%	N = 3	2018	ITO/PEDOT:PSS/perovskite/PCBM/BCP/Ag	ThMA_2_MA_*n*−1_Pb_*n*_I_3*n*+1_	PCE of around 90% retained under N_2_ atmosphere, 30 ± 10% relative humidity over a period of 1000 h	[164]
		17.3%	N = 5	2022	ITO/PEDOT:PSS/perovskite/Spiro-OMeTAD/Au	ThMA_2_MA_4_Pb_5_I_16_	PCE of around 92% retained under N_2_ atmosphere, over a period of 3600 h	[165]
		19.06%	N = 5	2020	ITO/PEDOT:PSS/perovskite/PC_61_BM/BCP/Ag	ThMA_2_FA_4_Pb_5_I_16_	PCE of around 96% retained under N_2_ atmosphere, 80 °C over a period of 576 h	[166]
	BEA	14.86%	N = 3	2019	FTO/compact TiO_2_ (c-TiO_2_)/Perovskite/Spiro-OMeTAD/Au	BEA_0.5_MA_3_Pb_3_I_10_	PCE of around 82% retained under ambient atmosphere, 35% relative humidity over a period of 2400 h	[167]
		16.1%	N = 4	2019	ITO/PEDOT:PSS/perovskite/PCBM/Al	BEA_2_MA_3_Pb_4_I_13_	PCE of around 83.1% retained under N_2_ atmosphere over a period of 2400 h	[168]
		17.39%	N = 3	2019	FTO/TiO_2_/perovskite/Spiro-OMeTAD/Au	(BEA)_0.5_Cs_0.15_(FA_0.83_MA_0.17_)_2.85_Pb_3_(I_0.83_Br_0.17_)_10_	PCE of around 90% retained under ambient atmosphere over a period of 2400 h	[169]
	PEA	14.6%	N = 5	2020	ITO/PEDOT:PSS/perovskite/PC_61_BM/BCP/Cu	PEA_2_MA_4_Pb_5_I_16_	–	[170]
		18.04%	N < 6	2019	ITO/PTAA/perovskite/PC_61_BM/PEI/Ag	PEA_2_MA_*n*−1_Pb_*n*_I_3*n*+1_	PCE of around 96.1% retained over a period of 8 months	[171]
		22.1%	N = 2	2024	FTO/TiO_2_/perovskite/PTAA/Au	PEA_3_Pb_2_I_7_	PCE retained 95% under ambient atmosphere, 50% relative humidity over a period of 1000 h	[172]
	ALA	14.4%	N = 10	2018	ITO/TiO_2_/perovskite/Spiro-OMeTAD/Au	ALA_2_(MA_0.14_FA_0.81_Cs_0.05_)_9_Pb_10_I_29_	PCE retained 90% under ambient and 85 °C over a period of 500 h	[48]
	PGA	15.20%	N = 3	2020	ITO/PTAA/perovskite/C_60_/Al	(PPA)_2_(CH_3_NH_3_)_2_Pb_3_I_10_	PCE retained 65% under ambient and 85 °C over a period of 95 h	[155]
Dion-Jacobson Perovskite	PDA	13.0%	N = 4	2018	ITO/PEDOT:PSS/perovskites/C_60_/BCP/Ag	PDAMA_n−1_Pb_n_I_3n+1_	PCE of around 90% retained under dark, 85% relative humidity over a period of 1000 h	[179]
		13.8%	N = 4	2020	ITO/SnO_2_/perovskite/Spiro-OMeTAD, Li TFSI/Ag	PDAFA_3_Pb_4_I_13_	PCE of around 86% retained under N_2_ atmosphere over a period of 1000 h	[180]
		14.16%	N = 5	2019	ITO/PEDOT:PSS/perovskite/PC_61_BM/LiF,AI	PDAMA_4_Pb_5_I_16_	XRD peak retained in normal under ambient atmosphere over a period of 32 days	[181]
		18.30%	N < 5	2022	FTO/ETL/perovskites/PCBM/Au	PDACs_x_MA_3−x_Pb_4_I_13_	PCE of around 95% retained under one-sun illumination over a period of 5000 h	[182]
	BDA	16.38%	N = 5	2019	ITO/PEDOT:PSS/perovskite/PC_61_BM(LiF,AI)	BDAMA_4_Pb_5_I_16_	XRD peak retained in normal under ambient atmosphere over a period of 32 days	[182]
		17.91%	N = 5	2019	ITO/PEDOT:PSS/perovskite/PC_61_BM/(LiF, Au)	BDAMA_n-1_Pb_n_X_3n+1_	PCE of around 84% retained under ambient atmosphere over a period of 1182 h	[183]
		17.34%	-	2023	ITO/(PTAA,PASP)/perovskite/C_60_/BCP/Ag	BDAMA_4_Pb_5_I_16_	PCE of around 80% retained under N_2_ atmosphere, one-sun illumination and 40 °C over a period of 800 h	[184]
	DMePDA	24.7%	N = 1	2024	glass/FTO/TiO_2_/perovskite/spiro-OMeTAD/Au	FA_0.85_MA_0.1_Cs_0.05_PbI_2.9_Br_0.1_/DMePDAI_2_	PCE of around 90% retained under N_2_ atmosphere, one illumination and 40 ºC over a period of 1000 h	[145]
	3AMP	12.04%	N = 4	2019	FTO/PEDOT:PSS/perovskite/fullerene (C_60_)/BCP/Ag	3AMP(MA_0.75_FA_0.25_)_3_Pb_4_I_13_	PCE of around 22% retained under ambient atmosphere, relative humidity and AM1.5G illumination over a period of 47.5 h	[185]
		16.25%	N = 4	2020	ITO/(PTAA/PFN)/perovskite/PC_61_BM/BCP/Ag	3AMP(MA_0.75_FA_0.25_)_3_Pb_4_I_13_	PCE of around 80% retained under ambient atmosphere, 45±5% relative humidity over a period of 35 days	[186]
		18.67%	-	2021	ITO/(NiO_*x*_/PTAA)/perovskite/PC_61_BM/BCP/Ag	3AMP(MA_0.75_FA_0.25_)_3_Pb_4_I_13_	PCE of around 90% retained under N_2_ atmosphere, 45±5% relative humidity and 85 °C over a period of 480 h	[187]
		20.4%	N = 5	2024	FTO/MeO-2PACz/perovskite/PC61BM/BCP/Ag	3AMPFAPb_2_I_7_	PCE of around 96% retained under N_2_ atmosphere, one-sun illumination over a period of 300 h	[188]
	4AMP	16.53%	N = 4	2020	ITO/TiO_2_/perovskite/Spiro-OMeTAD/Au	MAMPMA_3_Pb_4_I_13_	PCE of around 92% retained under 40% ~ 50% relative humidity over a period of 1000 h	[189]
		17.7%	–	2023	glass/ITO/PEDOT:PSS/4-AMPI_2_ + HPbI_3_/PVK/PCBM/BCP/Ag	4AMPPbI_4_	PCE of around 70% retained under N_2_ atmosphere over a period of 1200 h	[190]
	3AMPY	9.20%	N = 3 or 4	2019	FTO/PEDOT:PSS/perovskite/C_60_/BCP/Ag	3AMPYMA_3_Pb_4_I_13_	–	[191]
	4AMPY	5.69%	N = 3 or 4	2019	FTO/PEDOT:PSS/perovskite/C_60_/BCP/Ag	4AMPYMA_3_Pb_4_I_13_	–	[191]
Alternating Cations in the Interlayer space Perovskites	GA	7.26%	N = 3	2017	FTO/PEDOT:PSS/perovskite/PC_61_BM/Al	GAMA_3_Pb_3_I_10_	–	[195]
		18.48%	N = 3	2019	FTO/TiO2/perovskite/Spiro-OMeTAD/Au	(GA)(MA)_*n*_Pb_*n*_I_3*n*+1_	PCE of around 95% retained under 30% ~ 40% relative humidity and 25 °C over a period of 131 days	[196]
		19.18%	N = 5	2021	FTO/c-TiO_2_/perovskite/Spiro-OMeTAD/Au	(GA)MA_*n*_Pb_*n*_I_3*n*+1_	PCE of around 95% retained under ambient conditions, 30% ~ 40% relative humidity and (25 ± 5 °C) over a period of 123 days	[197]
		22.73%	N = 5	2024	ITO/NiO_x_/MeO-2PACz/perovskite/PEAI/PCBM/BCP/Ag	GA MA_5_Pb_5_I_16_	PCE of around 90.21% retained under room temperature (25 ± 5 °C), 50 ± 5% relative humidity over a period of 1200 h	z

Additionally, an example involving the length of the linear chain can be cited. As chain length increases, the material lattice structure becomes softer. This soft characteristic reduces the mechanical property strain, which decreases lattice mismatch and tilting angles, thereby enhancing structural stability. However, the wide spacing between RP perovskite lattices must be considered, as excessive spacing can degrade charge transport characteristics, leading to decreased efficiency. Therefore, selecting an appropriate length for the ligand is a crucial factor (Fig. 4f). In the study by Zheng et al*.*, devices composed of PA_2_(MA)_n−1_Pb_n_I_3n+1_ and BA_2_(MA)_n−1_Pb_n_I_3n+1_ (n = 3, 5, 7, 9, 11, and ∞) were analyzed, comparing the longest alkyl chain BA with the shorter chain propylammonium (PA). The PCE values observed from *J-V* curves for BA were 7.21% (n = 3), 11.28% (n = 5), 13.17% (n = 7), 14.99% (n = 9), 16.08% (n = 11), and 17.41% (n = ∞), which were lower compared to PA which showed efficiencies of 8.52% (n = 3), 12.04% (n = 5), 13.97% (n = 7), 15.22% (n = 9), 16.40% (n = 11), and 17.41% (n = ∞) as shown in Fig. 4g, h. These results demonstrate that as n approaches infinity, the quasi-2D perovskites behave similarly to high-efficiency 3D perovskites, and also that PA, with relatively shorter ligands, shows better efficiency. However, upon observing the PCE variation curves for device stability, after 1440 h of unsealed 2D perovskite devices under about 50% RH humidity aging, the normalized PCE of the MAPbI_3_ device rapidly reduces to 30%. Meanwhile, the PCE values for BA were 85% (n = 3), 80% (n = 5), 74% (n = 7), 66% (n = 9), 57% (n = 11), and 30% (n = ∞). For PA, the values were 82% (n = 3), 77% (n = 5), 70% (n = 7), 63% (n = 9), 55% (n = 11), and 30% (n = ∞). All values, except for n = ∞, indicated that shorter PA chains experienced smaller drops in PCE efficiency (Fig. 4i, j). This indicates that BA, with longer A′ cation chains, maintains stability better compared to perovskites with shorter A′ cation chains [156].

The experimental results align with the theory that shorter ligand cations increase rigidity, enhancing charge transport but reducing stability. Conversely, longer ligands offer greater flexibility, mitigating lattice tilt and maintaining stability over time. The graph in Fig. 4k, which measures and compares the Bohr radius of the lattice according to the length of the ligands, further details these trends. PEA exhibits a large Bohr radius (long ligand) and has a low dielectric constant that enhances exciton binding energy and stability. On the other hand, ethylammonium (EA) has a high dielectric constant, resulting in reduced exciton binding energy and relatively easier movement of electrons (Fig. 4k). Consequently, PEA shows opposite characteristics compared to EA, exhibiting behaviors influenced by the flexibility features mentioned earlier in RP [157].

RP perovskites incorporate a variety of A′ cations such as PA, BA, ThMA (2-thiophenemethylammonium), BEA (1-amino-3-butene), and PEA, each influencing the structure and thereby altering optoelectronic properties significantly (Fig. 4l) [158–172]. These variations in structure lead to changes in PCE and elucidate mechanisms of action, as detailed in Table 1.

## Dion-Jacobson (DJ) Perovskites

DJ and RP perovskites differ fundamentally in their use of spacer cations. DJ perovskites utilize divalent cations as spacers, while RP perovskites employ monovalent cations with a single ammonium group. This structural difference allows the alkyl ligands in RP perovskites to interact via vdW forces, creating considerable space that enables movement and flexibility. Conversely, DJ perovskites incorporate divalent cations with two functional groups each, which form direct bonds with the perovskite lattice, resulting in a more rigid structure devoid of intermediary vdW interactions (Fig. 2a). This significantly reduces the space available for alkyl ligand movement. Although one might expect better charge transport in DJ due to the reduced distance between perovskite lattices compared to RP, this perspective requires reconsideration. Observations from numerous experiments show that the PCE in DJ is lower than in RP, which necessitates examining the A′ cations in the DJ structure [173, 174].

**Fig. 5:**
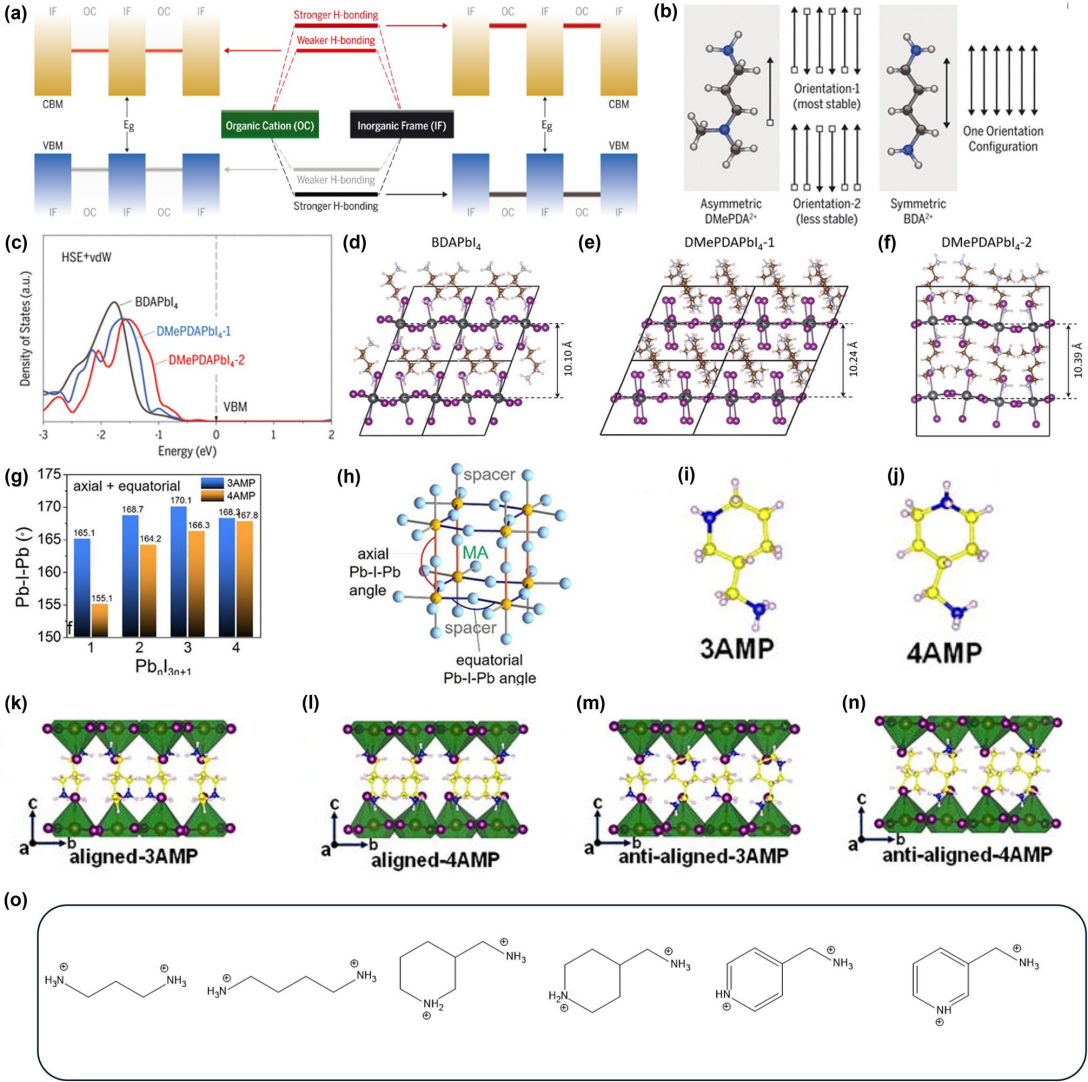
Dion-Jacobson perovskite (DJ perovskite) distortion affected organic cation in many parts of means. **a** Illustration of band offsets between [PbI_6_] planes and bulky organic cations with a weaker and stronger degree of H-bonding. For clarity, the inorganic framework orbital diagram is omitted in the middle of the panel. **b** Two possible arrangements of asymmetric DMePDA^2+^ cations and the sole arrangement of symmetric BDA^2+^ cations. **c** HSE + vdW calculated total DOSs of the organic cations in BDAPbI_4_, DMePDAPbI_4_-1 [with orientation-1 in **b**], and DMePDAPbI_4_-2 [with orientation-2 in **b**]. The VBMs were set to 0.0 eV. **d** Side view of the crystal structures of BDAPbI_4_, **e** DMePDAPbI_4_-1, and **f** DMePDAPbI_4_-2 single crystals. **a**–**f** are reprinted with permission from [176], copyright 2021 Science. **g** Average axial and equatorial angles for 3AMP and 4AMP. **h** Definition of axial and equatorial Pb–I–Pb angles. **g** and **h** are reprinted with permission from [177], copyright 2018 American Chemical Society. **i** Shape of 3AMP asymmetric cation. **j** Shape of 4AMP symmetric cation. **k** (3AMP)(MA)_3_Pb_4_I_13_ (3AMP) and **l** (4AMP)(MA)_3_Pb_4_I_13_ (4AMP) aligned conformations. **m** and **n** show their anti-aligned conformations, respectively. **i-n** are reprinted with permission from [178], copyright 2021 Applied Physics Letters. **o** Shape of each divalent organic cation PDA(1,3-propanediammonium), BDA(1,4-butanediammonium), 3AMP(3-(aminomethyl)piperidinium), 4AMP(4-(aminomethyl)piperidinium), 3AMPY(3-(aminomethyl)pyridinium), 4AMPY(4-(aminomethyl)pyridinium)

DJ perovskites follow a structure of A′A_(n-1)_B_(n)_X_(3n+1)_, which emphasizes hydrogen bonding between the organic A′ cation and the inorganic perovskite framework. While similar bonding occurs in RP perovskites, the rigid nature of DJ perovskites highlights these hydrogen bonds. It was believed that using divalent cations in DJ perovskites would reduce the spacer area compared to RP, potentially solving the problem of low carrier mobility in quasi-2D perovskites. However, this resulted in lower PCE, and such rationale alone cannot fully explain the low PCE observed in DJ structures. It suggests that the energy barrier between BX_6_ units, mediated by A′ cations, remains unresolved. This interpretation can be substantiated by band offsets resulting from different dielectric constants between A′ cations and BX_6_ units. Typically, basic perovskite structures are formed with a conduction band minimum (CBM) and valence band maximum (VBM), with excitons confined within the inorganic frame. In quasi-2D perovskites, the addition of A′ cations introduces different offsets when these materials meet, creating a potential difference that segregates free electrons and holes, facilitating out-of-plane charge transport along the perovskite structure. Thus, optimizing these band offsets to maximize the effects of charge transport is crucial, with the strength of hydrogen bonding playing a significant role. Adjusting band offsets through the strength of hydrogen bonds, weak hydrogen bonding tends to place the bulky A′ cations at a high-energy position within VBM of the BX_6_ inorganic frame [175]. Therefore, DJ perovskites with weak hydrogen bonding exhibit improved hole transport (Fig. 5a). However, weak hydrogen bonding indicates relatively unstable perovskites, necessitating further research into metastable DJ perovskites.

To further leverage the efficiencies of metastable DJ perovskites, Zhang et al*.* compared N,N-dimethyl-1,3-propane diammonium (DMePDA^2+^) and 1,4-butane diammonium (BDA^2+^). DMePDA^2+^, being an asymmetric molecule, allows for freely formed head or tail configurations, facilitating diverse H-bonding orientations and thus enhancing hole transport with various band offsets. In contrast, the symmetric BDA^2+^ has a fixed orientation, making it challenging to create metastable states (Fig. 5b). Figure 5c shows the results from density functional theory (DFT) calculations on BDAPbI_4_, DMePDAPbI_4_-1, DMePDAPbI_4_-2 templating samples (Fig. 5d–f), revealed that BDAPbI_4_ has the shortest Bohr radius at 10.10 Å, suggesting that while it is structurally stable due to its symmetric orientation, it may not effectively enhance VBM, potentially leading to suboptimal efficiency. Conversely, DMePDAPbI_4_-2, with the longest Bohr radius at 10.39 Å, suggests potential improvements, with calculations of the states of C, N, and H atoms showing increased efficiency in out-of-plane hole transport compared to BDAPbI_4_. Consequently, the FA_0.85_MA_0.1_Cs_0.05_PbI_2.9_Br_0.1_-based perovskite demonstrated a PCE of 20.9%, whereas the DMePDAI_2_-modified perovskite achieved a maximum PCE of 24.7%, indicating an increase of 3.1% in efficiency. Following the ISOS-L-1 stability protocol, when subjected to maximum power point tracking (MPPT) under conditions of ~ 40 °C in an N_2_ environment, the unmodified perovskite exhibited a 43% drop in PCE after 1000 h, whereas the DMePDAI_2_-modified perovskite showed only a 10% decline, demonstrating a positive effect on stability [176].

The Pb-I-Pb angle also impacts out-of-plane transport. Mao et al*.* investigated the PCE of DJ perovskites made with 3-(aminomethyl)piperidinium (3AMP) and 4-(aminomethyl)piperidinium (4AMP) as the A′ cation depending on the Pb-I-Pb angle. By calculating the average Pb-I-Pb of the axial and equatorial parts of the perovskite by adjusting the *n* value from 1 to 4, the results showed that the Pb-I-Pb angle of 4AMP increased with increasingly higher *n* values, but the Pb-I-Pb angle of 3AMP was not constant. In addition, the average value of 3AMP was more ideal than that of 4AMP in most cases (Fig. 5g). The reason for this is thought to be that because of the structure of 3AMPs, there is a variety of H-bonding. And here it is necessary to know the meaning of axial and equatorial in a growth of perovskite lattice. In Fig. 5h, an illustration of each Pb-I-Pb angle is shown, where axial means along the longest crystallographic axis and equatorial means along the inorganic plane. These Pb-I-Pb angles are also closely related to the hydrogen bonding mentioned earlier, particularly, the equatorial part of 3AMP is less affected by hydrogen bonding compared to 4AMP, which explains why 3AMP is less tilted than 4AMP [177].

However, 3AMP is attached to the meta position of the piperidine, making it an asymmetric cation. In contrast, 4AMP is attached to the para part, rendering it a more symmetric cation then 3AMP (Fig. 5i, j). However, as discussed in the previous section, due to various hydrogen bonding interactions, the average value of the Pb-I-Pb angle in 3AMP is closer to the ideal 180°. To understand in more detail how this A′ cation is formed in the perovskite, Wang et al*.* compared the aligned and anti-aligned structures (Fig. 5k–n). The perovskites aligned in the ab-plane are categorized as aligned or anti-aligned depending on whether they match the backbone structure. Furthermore, the degree of this alignment influences their physical and chemical properties, where calculations have shown differences in the bandgap. Such subtle differences cannot be precisely measured with equipment like XRD, therefore, we used the Vienna Ab initio simulation package (VASP) for static electronic structure calculations and Ab initio molecular dynamics (AIMD) simulations to define the aligned structures of the cations. The calculated aligned structure (Fig. 5k, l) had a bandgap of 1.42 eV for 3AMP and 1.86 eV for 4AMP, respectively, while the anti-aligned structure (Fig. 5m and n) had a bandgap of 1.83 eV for 3AMP and 1.89 eV for 4AMP, respectively. This variation is related to hydrogen bonding lengths, where the respective lengths were 2.64 Å for 3AMP and 2.79 Å for 4AMP in the aligned structure, respectively, and 2.60 Å for 3AMP and 2.73 Å for 4AMP in the anti-aligned structure, respectively, indicating that on average, the hydrogen bonding lengths were shorter in the anti-aligned structure. This is in contrast to the bandgap, and considering the previous hydrogen bonding characterization, it can be observed that the anti-aligned structure has stronger hydrogen bonding. Thus, observing that asymmetric 3AMP achieves a closer-to-ideal 180° Pb-I-Pb angle compared to symmetric 4AMP, which would theoretically be expected to exhibit this ideal angle, highlights the crucial role of hydrogen bonding in attaining the ideal Pb-I-Pb angle [178].

For similar reasons, when comparing 3-(aminomethyl)pyridinium (3AMPY) and 4-(aminomethyl)pyridinium (4AMPY), 3AMPY demonstrates a more ideal 180° Pb-I-Pb angle along the equatorial axis. The key structural difference between AMP and AMPY is that AMP features a piperidinium ring, whereas AMPY contains a pyridinium ring. In the pyridinium structure, π bonding within the benzene ring shortens the C–C bonds, which reduces the interlayer spacing and decreases the dielectric mismatch between the inorganic layer and the organic spacer. Due to these characteristics, AMPY with a pyridinium structure, compared to AMP with a piperidinium structure, results in a rise in the dielectric constant of the interlayer space and greater delocalization of the positive charge on the aromatic ring [191]. The more PCE and stability of PDA (1,3-propanediammonium), BDA, 3AMP, 4AMP, 3AMPY, and 4AMPY are summarized in Table 1, alongside those of RP perovskites (Fig. 5o) [179–191].

## Alternating Cations in the Interlayer Space (ACI) Perovskites

The A′ cation in RP perovskites is a monovalent cation that adheres to each inorganic layer, reducing strain through the flexible nature of ligands attached to each cation, thereby providing structural stability. However, in contrast to DJ perovskites, this leads to increased vdW spacing between BX_6_ inorganic structures in RP perovskites. Conversely, while it was initially believed that DJ perovskites would facilitate charge transport due to the closer spacing between BX_6_ structures, it was found that the mechanisms in DJ perovskites do not support this advantage over RP perovskites [158–172]. Nonetheless, it was also observed that excessive ligand length in RP phases leads to a decrease in optoelectronic properties [179–191]. Consequently, ongoing optimization for each 2D structure is being pursued.

**Fig. 6:**
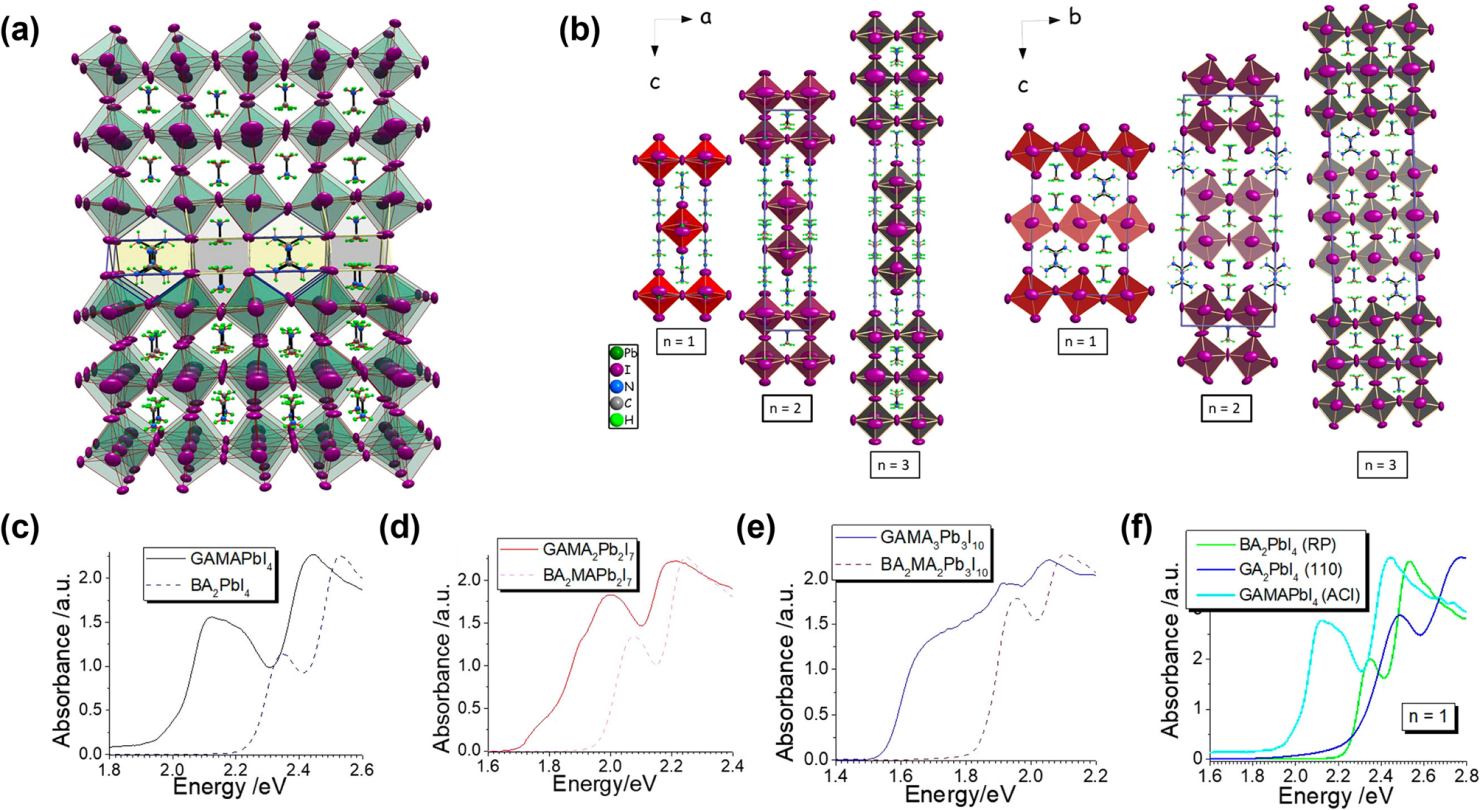
Alternating Cations in the Interlayer space (ACI) Perovskites distortion affected by organic cation. **a** Typical lattice structure of (GA(MA)_*n*_Pb_*n*_I_3*n*+1_ ACI perovskites. **b** Unit cells of (GA)(MA)_*n*_Pb_*n*_I_3*n*+1_ observed along the b-axis and a-axis, divided into categories n = 1–3. **c–e** Optical absorption spectra of the (GA)(MA)_*n*_Pb_*n*_I_3*n*+1_ (*n* = 1–3) perovskites. **f** Selected absorption spectra of *n* = 1 member of the ACI perovskite GAMAPbI_4_, the RP perovskite BA_2_PbI_4_, and the (110)-cleaved perovskite GA_2_PbI_4_ highlighting the importance of the perovskite structure-type on the optical properties of the materials. **a**–**f** are reprinted with permission from [195], copyright 2017 American Chemical Society

Meanwhile, the alternating cations in the interlayer space (ACI) structure, which incorporates both DJ and RP structures in forming 2D perovskite structures, has begun to gain attention. Kanatzidis et al. first reported ACI phase PSCs based on alternating arrangements of guanidinium (GA, C(NH_2_)_3_) and MA (CH_3_NH_3_). The ACI structure aims to harness the advantage of DJ’s narrow spacer area while leveraging the stable characteristics of RP for enhanced stability and efficiency. Guanidinium (GA) was employed as the primary A′ cation in the ACI structure. The addition of GA, which minimally distorts the BX_6_ inorganic layer structure, is suggested to result in a narrower bandgap compared to RP perovskites. The unusual ordering of GA and MA cations occupies the spacer area (Fig. 6a), with the relatively large GA at the edges and the smaller MA in the center, causing distortion. When viewed along the *a-*axis, the* a*-axis and *c*-axis of the (C(NH_2_)_3_)(CH_3_NH_3_)_n_Pb_n_I_3n+1_ (GAMA_n_Pb_n_I_3n+1_) perovskite structure are *a* = 6.4286 Å, *b* = 12.4577 Å (n = 1),* a* = 6.3729 Å, *b* = 12.5435 Å (n = 2),* a* = 6.3520 Å, *b* = 12.4378 Å (n = 3), showing minimal changes. However, when viewed along the b-axis, the *c*-axis length is* c* = 18.8258 Å, *c* = 31.376 Å, and *c* = 43.970 Å, extending more than twice the ideal unit cell length (Fig. 6b). This growth direction leads to a needle-shaped morphology, and strong octahedral tilting is observed along the b-axis. In contrast, RP phases exhibit distortion along both the* b* and *a* axes, suggesting that ACI structures with distortion along only one axis have less overall distortion [192].

Less distortion is associated with a narrower bandgap, as supported by the symmetry observed in perovskites with odd *n* values (*n* = 1, 3) showing mirror symmetry centered on the A′ cation, whereas even *n* values (*n* = 2) exhibit asymmetry. This symmetry loss in the perovskite lattice broadens the bandgap, explaining why the more distorted RP structures exhibit a wider bandgap than ACI structures [193]. Observing the photophysical properties of GA_2_PbI_4_—a highly distorted ⟨110⟩-sliced perovskite—via absorption spectra, GA_2_PbI_4_ has the broadest bandgap with *E*_g_ = 2.49 eV [194]. Examining bandgaps across perovskites with GA cations, ACI perovskites display bandgaps of *E*_g_ = 2.27 eV for n = 1, *E*_g_ = 1.99 eV for n = 2, and *E*_g_ = 1.73 eV for *n* = 3, whereas BA_2_PbI_4_ RP perovskites show a larger bandgap (Fig. 6c-f). Devices made from quasi-2D perovskites with the (C(NH_2_)_3_)(CH_3_NH_3_)_n_Pb_n_I_3n+1_ structure achieved an open-circuit voltage (*V*_oc_) of 0.988 V, a short-circuit current density (*J*_sc_) of 9.84 mA cm^−2^, a fill factor (FF) of 79.68%, and a PCE of 7.26% [195].

Kanatzidis’ findings opened new possibilities for ACI structures, and the PCE efficiencies of quasi-2D perovskite-based devices achieved in various studies are summarized in Table 1 [196, 197].

Despite these advancements, structures incorporating components other than GA have yet to be developed. This limitation stems from the incomplete understanding of the exact mechanisms underlying the ACI crystal structure. Future research to clarify these mechanisms is expected to enable the use of alternative A′ cations, potentially overcoming material limitations and achieving higher efficiencies. This represents a forward-looking direction for researchers studying quasi-2D perovskites.

## Additive Engineering

**Fig. 7:**
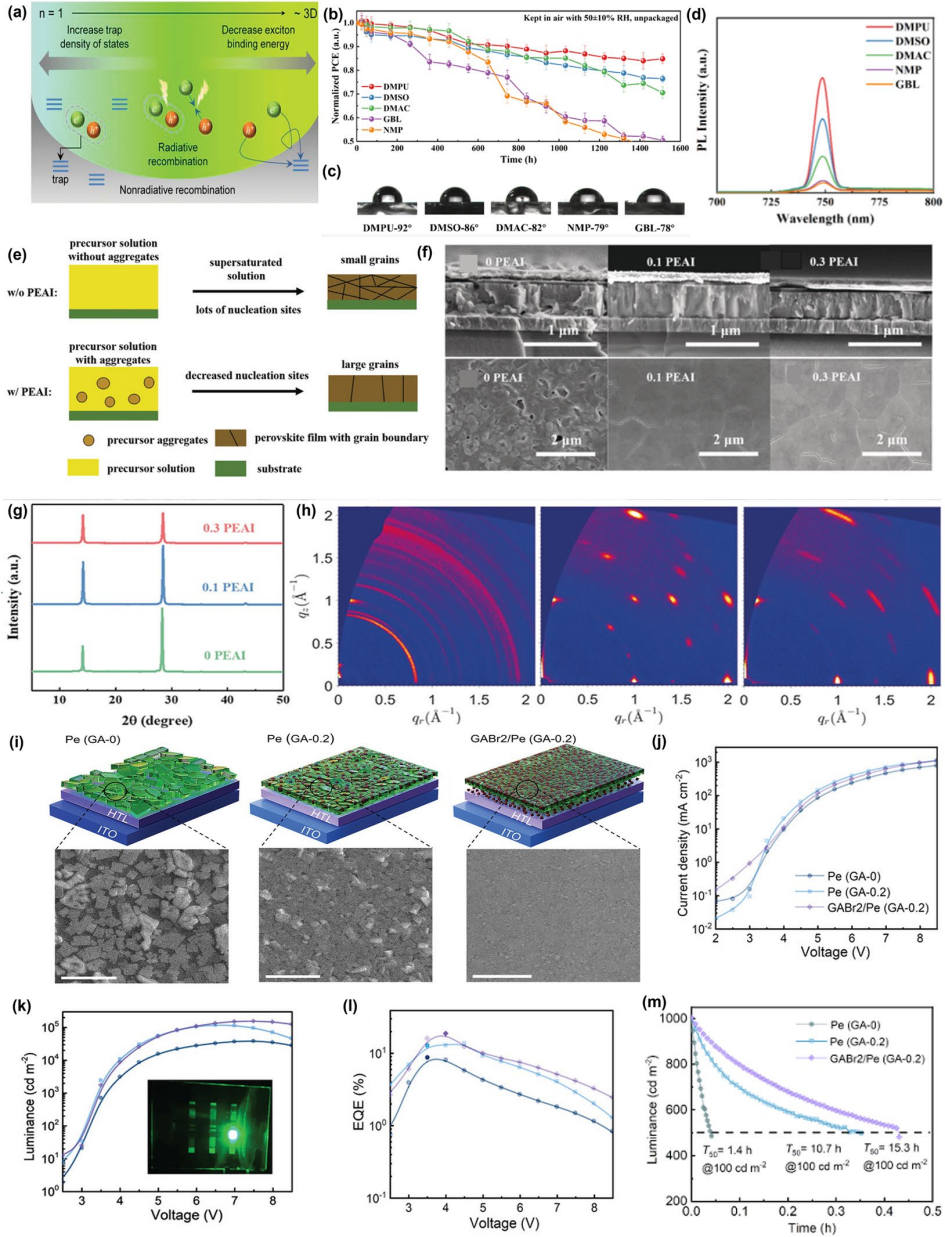
Modification method about grain of 2D perovskite by additive is revealed throughout enhanced efficiency. **a** Recombination tendency between large n and small n. **a** is reprinted with permission from [198], copyright 2021 Advanced Functional Materials. **b** shows PCE decay measurements based on unpackaged devices prepared by different solvents in air (average values were obtained based on 3 devices in each condition).** c** Each different solvent has different contact angle (the more hydrophobic, the more large angle). **d** PL spectra of the perovskite films prepared with different solvents. **b**-**d** are reprinted with permission from [205], copyright 2022 Molecules. **e** Schematic Illustration of the formation of the 2D perovskite film without or with PEAI addition. **f** Side & Top view of the grain of SEM images. **g** XRD patterns of BA_2_MA_4_Pb_5_I_16_ perovskite films with various amounts of PEAI. **h** 2D GIWAXS patterns of BA_2_MA_4_Pb_5_I_16_ perovskite films containing (From the left, 0 PEAI, 0.1 PEAI and 0.3 PEAI). **e**-**h** are reprinted with permission from [211], copyright 2019 Angewandte Chemie. **i** Schematic diagrams and corresponding top-view SEM images of the perovskite films produced without additive, Pe (GA-0); with GABr doping, Pe GA-0.2; with interface layer, GABr_2_/Pe (GA-0.2). **j** *J-V* curve for each sample: Pe (GA-0), Pe (GA-0.2), GABr_2_/Pe GA-0.2. **k** Luminance–voltage (*L* − *V*) for each sample: Pe (GA-0), Pe (GA-0.2), GABr_2_/Pe GA-0.2. **l** EQE − voltage (EQE − V) for each sample: Pe (GA-0), Pe (GA-0.2), GABr_2_/Pe GA-0.2. **m** Operational lifetime characteristics of PeLEDs based on Pe (GA-0), Pe GA–0.2 and GABr_2_/Pe (GA-0.2). (**i**-**m**) are reprinted with permission from [223], copyright 2022 Advanced Functional Materials

Additive engineering traditionally involves the incorporation of additives such as MACl to reduce trap density and enhance the crystallinity of perovskite lattices. Over time, numerous optimizing techniques have emerged to further improve efficiency. For instance, adjusting the *n* values in 2D perovskites influences trap densities (Fig. 7a). Higher *n* values typically correlate with larger grain sizes and reduced trap densities but also lower exciton binding energies, which can diminish charge and carrier confinements. Conversely, lower *n* values increase exciton binding energy, which is beneficial but can also increase trap density, leading to nonradiative recombination [196, 198, 199]. Therefore, numerous factors must be considered when optimizing quasi-2D perovskites. Unlike 3D perovskites, quasi-2D perovskites require distinct optimization approaches. This section introduces various unique optimization methods.

The choice of precursor and solvent is crucial in controlling these parameters, making solvent selection a vital part of the fabrication process. This applies to both 3D and 2D perovskites. In 3D perovskite solvent engineering, perovskite formation involves the creation of intermediate phases, such as HI, MA, and MAAc intermediates, which are then effectively reconfigured into the perovskite structure through antisolvent and annealing processes [200]. In quasi-2D perovskites, this process is largely similar. However, the critical focus in quasi-2D solvent engineering is promoting vertical crystal growth. Thus, unlike in 3D perovskites, quasi-2D solvent engineering requires slowing down the crystallization rate of the solvent and minimizing nucleation growth as much as possible [201–204]. This approach effectively promotes vertical crystal growth, which we demonstrate through the following experiments. Su et al*.* demonstrated that various solvent-assisted methods could enhance stability. By modifying the solvent, devices based on dimethylpropyleneurea (DMPU) exhibited the best stability, maintaining 85% of their initial PCE after 1512 h in an air atmosphere (RH 50 ± 10%) without encapsulation. In comparison, devices using DMSO and dimethylacetamide (DMAC) retained 76% and 71% of their initial PCE, respectively. Devices with N-methyl-2-pyrrolidone (NMP) and γ-butyrolactone (GBL) showed a rapid decline in performance, with the PCE dropping to 50% after about 1512 and 1360 h, respectively. DMPU-based 2D perovskites also had the largest contact angle, indicating hydrophobicity plays a role in preventing perovskite degradation (Fig. 7b, c). This investigation revealed that the quality of 2D perovskite films and device performance corresponded directly to the Lewis basicity of the solvents used. An increase in the Lewis basicity of these solvents led to a marked slowdown in the crystallization rate. This effect can be attributed to the tendency of Pb^2+^ (acting as a Lewis acid) to form bonds with Lewis bases, indicating solvents with higher Lewis basicity competed more effectively with I^−^ for coordination sites surrounding Pb^2+^. Consequently, the stronger a solvent's Lewis basicity, the more potent its binding capacity with Pb^2+^, resulting in a delayed release of Pb^2+^ during crystallization. In addition, the high PL intensity in DMPU-based perovskites indicates superior light absorption capabilities, enabling more efficient carrier generation (Fig. 7d). This suggests better crystalline quality, supported by lower defect densities and fewer trap states, leading to enhanced spontaneous radiative recombination. Comparatively, the perovskite film based on GBL exhibited the poorest photophysical properties, highlighting the significant role of solvent choice in film quality and performance. The observed variations across different solvents also indicate the critical influence of Lewis basicity in the crystallization process of 2D perovskites, where higher Lewis basicity correlates with improved film quality and device performance [205, 206].

Another additive method is to add an additional A′ cation to the 2D perovskite. This is a unique additive method specific to 2D materials, where introducing and additional A′ cation, different from the main A′ cation, induces aggregation within the precursor. During film formation via this approach, prefer orientation growth occurred, allowing the enhanced stability of PCSs [207–210]. To investigate this further, Lian et al*.* experimented that adding a second spacer cation (SSC^+^) to the precursor while utilizing PEA^+^ as a main A′ cation of a perovskite based on BA_2_MA_n-1_Pb_n_I_3n+1_ perovskite. As a result, the unsealed device demonstrates good moisture stability, maintaining about 90% of its initial efficiency after 1000 h of air exposure (RH = 25 ± 5%). Additionally, it achieved a maximum PCE of 14.09%, the highest recorded for 2D BA_2_MA_n-1_Pb_n_I_3n+1_ (n = 5). Adding PEAI as a precursor aggregate induced nucleation and decreased nucleation sites, resulting in larger grain sizes. This created a noticeable difference in grain size between samples without and with PEAI (Fig. 7e). To see the effect of PEAI (x = 0, 0.1, or 0.3) aggregates, devices containing the mixture of BAI:PEAI:MAI:PbI_2_ = 2(1-x):2x:4:5 were examined by scanning electron microscope (SEM) to observe the side and top sections (Fig. 7f). Without PEAI addition, cavities were seen on the side and pinholes were observed on the top. However, no cavities were observed on the side of the film with PEAI addition, and the film viewed from the top became smoother with no pinholes. The 0.1 PEAI addition film recorded a grain size of almost 1 μm, with minimum grain boundaries. XRD (Fig. 7g) and GIWAXS (Fig. 7h) further quantified film quality, showing that PEAI positively effect on the film formation. In XRD, the angles of 14.1° and 28.3° were observed as (111) and (202), respectively, with the (111) peak increasing intensity, especially at 0.1 PEAI, which showed the highest intensity of 6.7 × 10^4^ cps and was the most crystalline. However, excessive additive, such as 0.3 PEAI, ruined the topology of the film with low (111) intensity. GIWAXS also showed that without PEAI addition, the scattering peaks were ring-like. Here, the excess of 0.3 PEAI caused the Bragg spots to span a slightly wider polar angle. Therefore, in this experiment, the 0.1 PEAI with the most optimal Bragg spots formed the best quality film [134, 211, 212].

Additionally, this SSC^+^ can also be applied to LED devices. Quasi-2D perovskites inherently possess high exciton binding energy due to the unique quantum well structure of the device, which reduces non-radiative recombination. This can lead to excellent EQE performance, thus positively impacting LED devices [213–218]. Based on different n-values, domains are divided, and the rapid energy transfer process between these domains can lead to carrier localization and accumulation in higher n domains, which enhances the luminescence of the emitter. However, low n domains provide stability but lower efficiency. Therefore, adjusting the n-value is proposed as a means to find a compromise between lifetime and electroluminescence performance [219–221].

Kong et al. experimented with the quasi-2D perovskite precursor BA_2_Cs_n-1_Pb_n_Br_3n+1_ by adding GA^+^ ions to the interface between the hole transport layer (HTL) and assisted nucleation, achieving an external quantum efficiency (EQE) of over 20% and a luminance up to 100,000 cd m^−2^. In Fig. 7I, GABr was added to each perovskite to replace BABr, resulting in the creation of Pe (GA-0), Pe (GA-0.2), and GABr_2_/Pe (GA-0.2). By adding GA, they divided into GA-0 and GA-0.2, and GABr_2_/Pe (GA-0.2) was prepared with a thin interlayer on the perovskite, acting as a seed to allow vertical growth of the perovskite when GA^+^ was added.

As a result, SEM images of Pe (GA-0) and Pe (GA-0.2) films showed numerous pinholes, grain boundary gaps, inhomogeneities, and grain sizes ranging from approximately 50 to 350 nm. However, the interlayered GABr_2_/Pe (GA-0.2) formed a highly dense film with a grain size of about 10 to 30 nm. Unlike PSCs, the quantum well structure reduces grain size, which, in turn, increases exciton binding energy due to smaller grains, resulting in a highly beneficial effect on LEDs with high EQE. The reason for the formation of such a dense film without pinholes is twofold. First, GA^+^, a dication, has stronger hydrogen bonding within the perovskite compared to BA^+^, a monoammonium cation, thus facilitating the growth of dense and uniform perovskite films. GA^+^ reacts with Pb^2+^ to form an intermediate during film formation, which slows down the crystallization of BA^+^, promoting vertical growth. The GA^+^ ions at the interface serve as seeds, providing numerous nucleation sites that help form dense grains.

Kong et al. observed the *J-V* curve, luminance-voltage, and EQE-V graph for each of the films produced by these two effects. In the *J-V* curve, Pe (GA-0.2)-based LEDs were more efficient than Pe (GA-0)-based LEDs, likely due to the decreased resistance of the perovskite film as grain size increased. In GABr_2_/Pe (GA-0.2), aside from grain size, the reduction of pinholes and improvement in film uniformity led to a slight reduction in current density over the 3.5–7.5 V range, achieving an improved charge balance (Fig. 7j).

In terms of luminance, the GABr-modified LED exhibited a lower turn-on voltage (*V*_on_) of 2 V and reduced leakage current. This was attributed to the improved morphology of the film. Once V_on_ was surpassed, radiative recombination was observed, reaching the highest luminance of 105,428 cd m^−2^ for GABr_2_/Pe (GA-0.2)-based LEDs, approximately four times higher than that of Pe (GA-0)-based LEDs (27,228 cd m^−2^) (Fig. 7k).

Finally, the average EQE of Pe (GA-0), Pe (GA-0.2), and GABr_2_/Pe (GA-0.2)-based LEDs was 8.8%, 13.7%, and 18.7%, respectively, with the optimized GABr_2_/Pe (GA-0.2) achieving a maximum EQE of 20.1% (Fig. 7l). Regarding stability, the half-lifetime (T50) in GABr_2_/Pe (GA-0.2) was 15.3 h at an initial luminance of 100 cd m^−2^, compared to 10.7 h for Pe (GA-0.2) and 1.4 h for Pe (GA-0)-based LEDs, demonstrating remarkable stability (Fig. 7m). This effect can be attributed to the improvement in film quality, interfacial contact, and charge balance [222].

Similarly, the quality of quasi-2D perovskites can be improved by adding additives to 3D perovskites, and there may be more ways to do so. While 3D perovskite structures focus on improving the inorganic framework of the material, quasi-2D perovskites allow for optimization of the A′ cation, which can significantly increase the exciton binding energy. In quasi-2D perovskites, considering the n values, which define the number of inorganic layers between organic spacers, is crucial to fully leverage the benefits of 2D perovskites. This structural variation influences the electronic properties and enhances device performance by managing the dimensional confinement of excitons [223–231]. Matching the *n* value may be challenging for solution-processed perovskite, so using such additives can enhance efficiency. Further study of the additive methods should be studied to better understand their impact on the stability and performance of quasi-2D perovskites.

## Conclusion and Outlook

In conclusion, quasi-2D perovskite represents a promising advancement in the field of perovskite materials for applications such as LEDs and PSCs, due to their ambient environment-resistant characteristics and high exciton binding energy compared to 3D perovskite. These characteristics, attributed to the distinct A′ cations used in quasi-2D structures, suggest their potential as a viable replacement for 3D perovskites, which are more sensitive to environmental conditions. This makes quasi-2D perovskites attractive for commercialization. However, despite their appeal, the limitations of 2D structures are clearly present. Despite their enhanced stability, quasi-2D perovskites often exhibit lower PCE compared to their 3D counterparts due to the inherent trade-off between stability and efficiency in structures where each barrier layer is formed by different A′ cations. Strategies such as adjusting the n value to create 3D-like quasi-2D perovskites or optimizing the Pb-I-Pb angle through A′ cation modification show potential, but further research is needed to balance stability and efficiency effectively. Additionally, integrating heterostructure approaches that layer 2D and 3D perovskites can also harness the efficiency of 3D structures while maintaining the stability of 2D layers, proposing potential avenues for future development [232]. Furthermore, various improvement methods utilizing quasi-2D perovskites not only have applications in LEDs and PSCs but can also be extended to devices like memristors, which are used in computation-intensive Neuromorphic hardware. This broadens the potential applications of quasi-2D perovskites, opening up new possibilities in high-performance electronic devices. Therefore, it is necessary to understand the detailed mechanisms and structures of quasi 2D perovskites, specifically for RP and DJ perovskites. Such knowledge can provide insights into overcoming existing challenges. This review paper aims to cover the most fundamental aspects of these perovskite structures, offering essential knowledge to a broad audience. Additionally, by discussing the more details of 2D perovskites, such as Lewis base, SSC^+^, and others through additive engineering, it suggests a complementary method to 2D perovskites and proposes directions for future research.
